# Supplementary figures and images for: Association between demographic, clinical characteristics and severe complications by SARS-CoV-2 infection in a community-based healthcare network in Chile
Source: PLoS One. 2024 Dec 30;19(12):e0314376. doi: 10.1371/journal.pone.0314376 (PMC11684639; doi:10.1371/journal.pone.0314376)

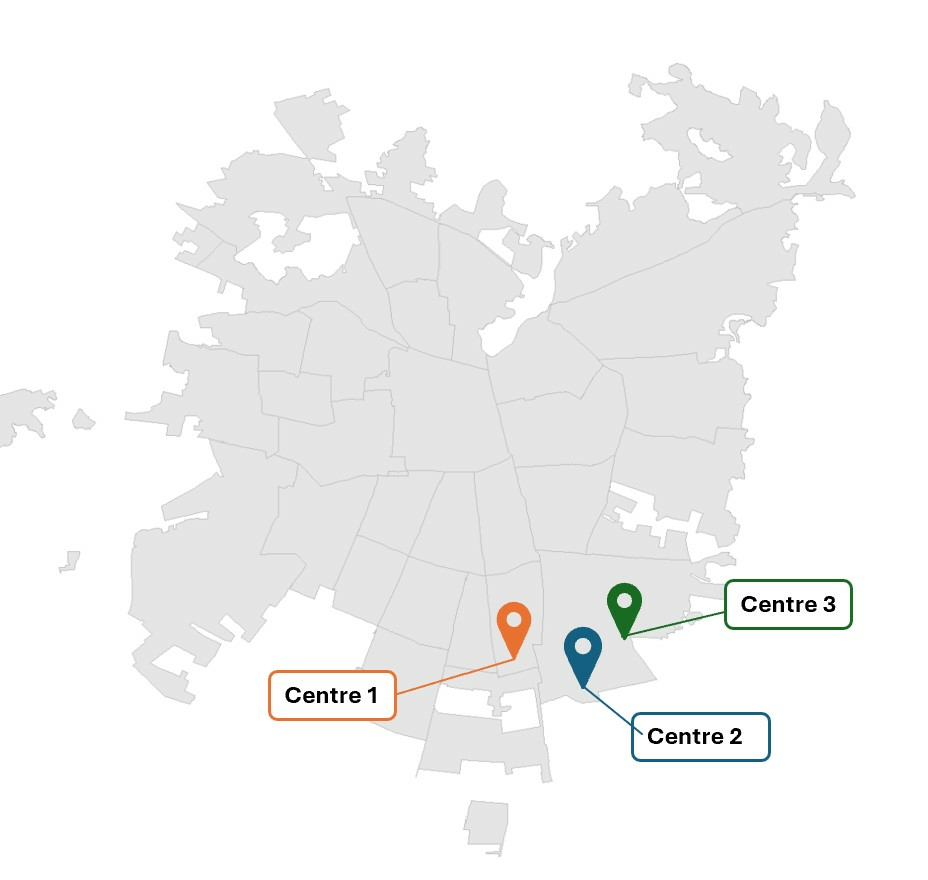

Supplement: S1 Fig — (TIF) [file pone.0314376.s001.tif]
