# Supplementary material for: Association between demographic, clinical characteristics and severe complications by SARS-CoV-2 infection in a community-based healthcare network in Chile
Source: PLoS One. 2024 Dec 30;19(12):e0314376. doi: 10.1371/journal.pone.0314376 (PMC11684639; doi:10.1371/journal.pone.0314376)

S4 Fig. Kaplan-Meier failure functions

1. **Age**


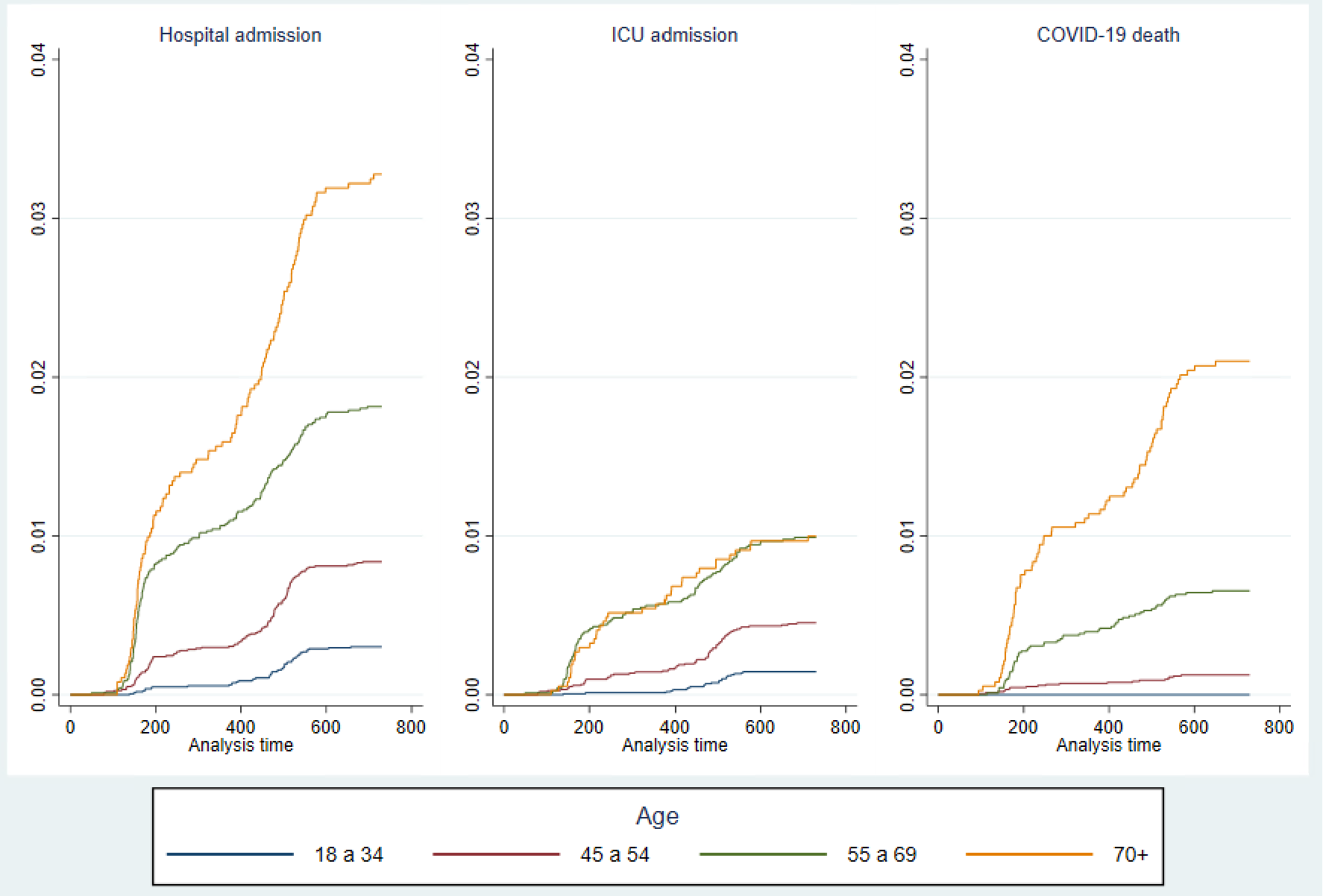


1. **Sex**


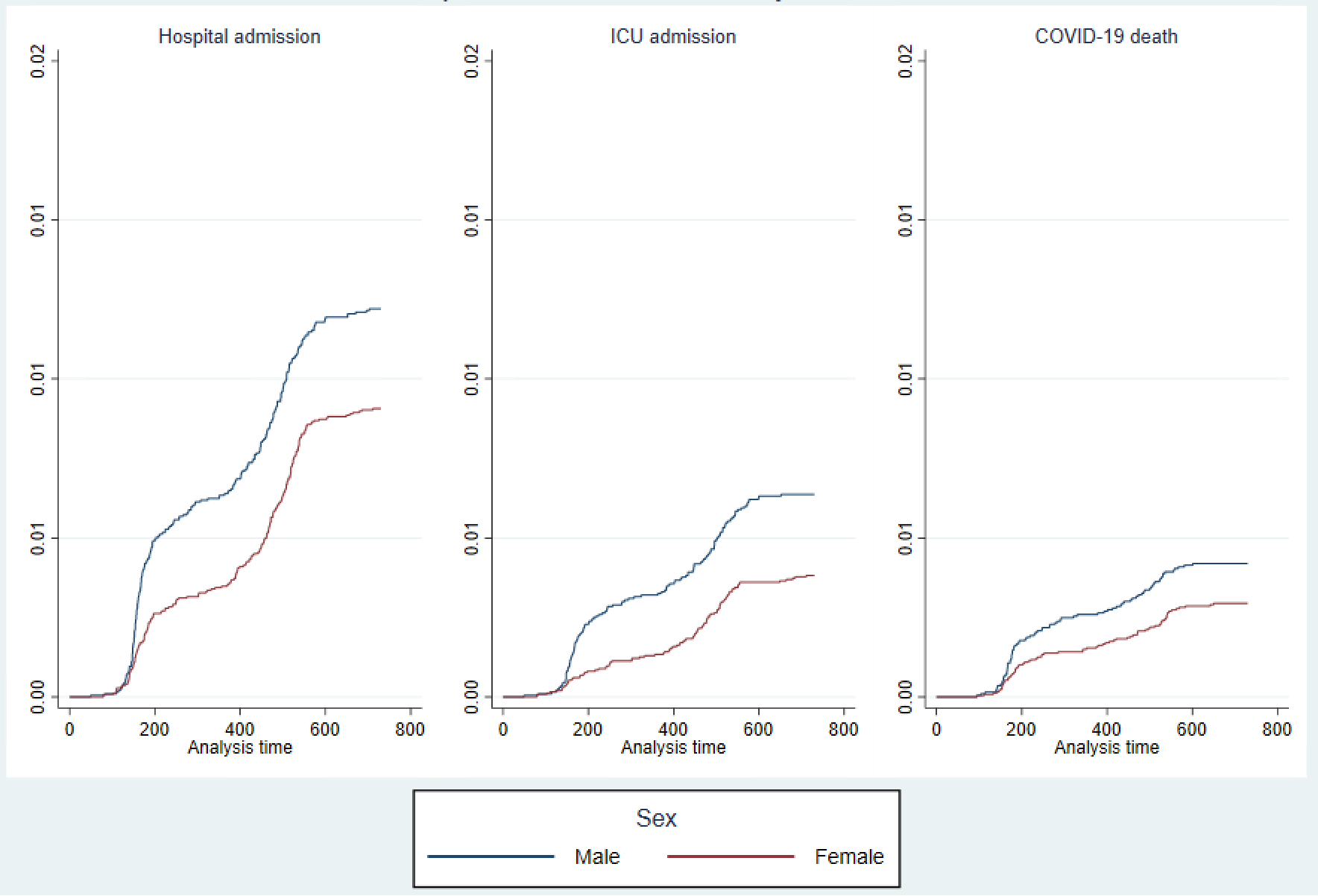


1. **Fonasa type**


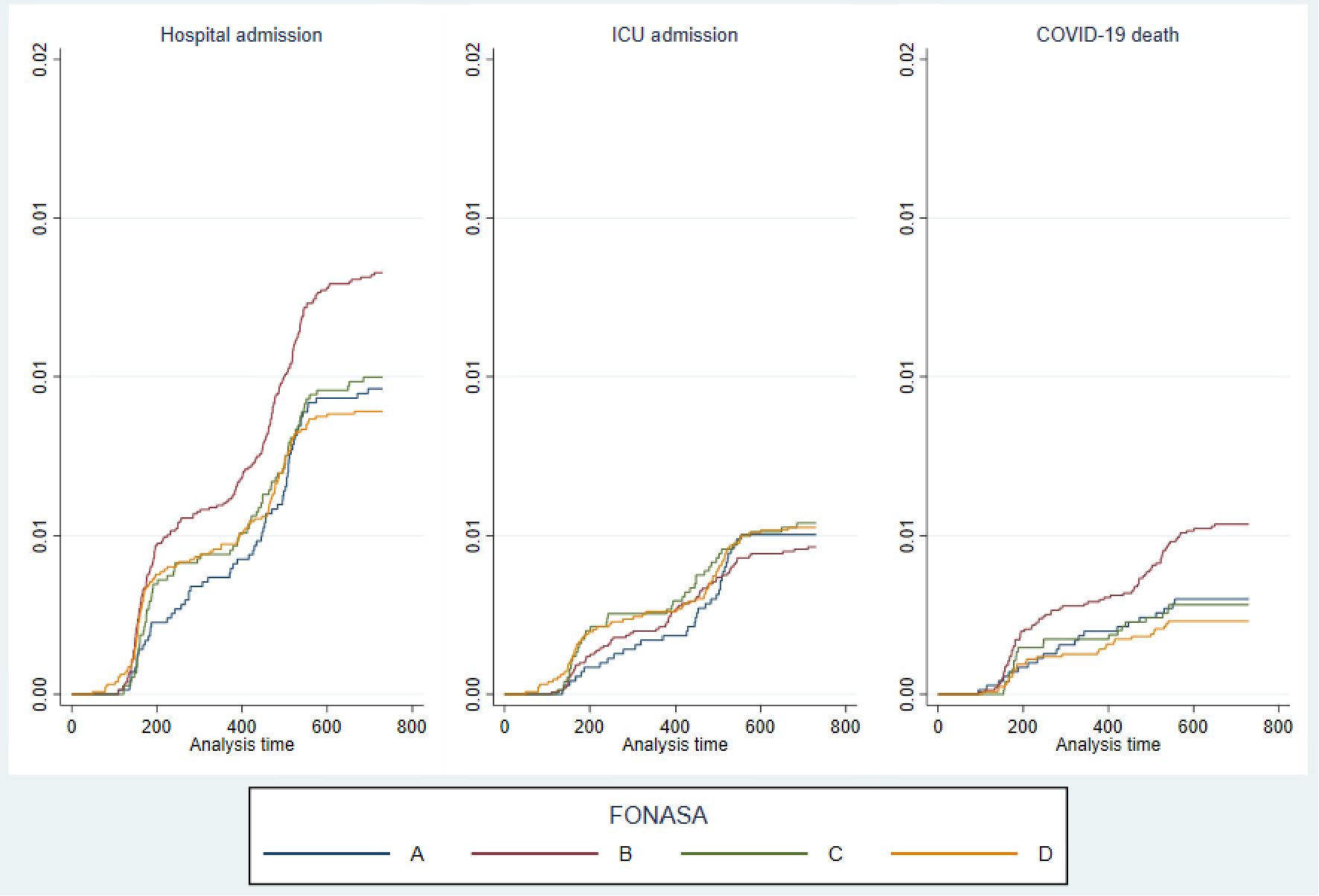


1. **Hypertension**


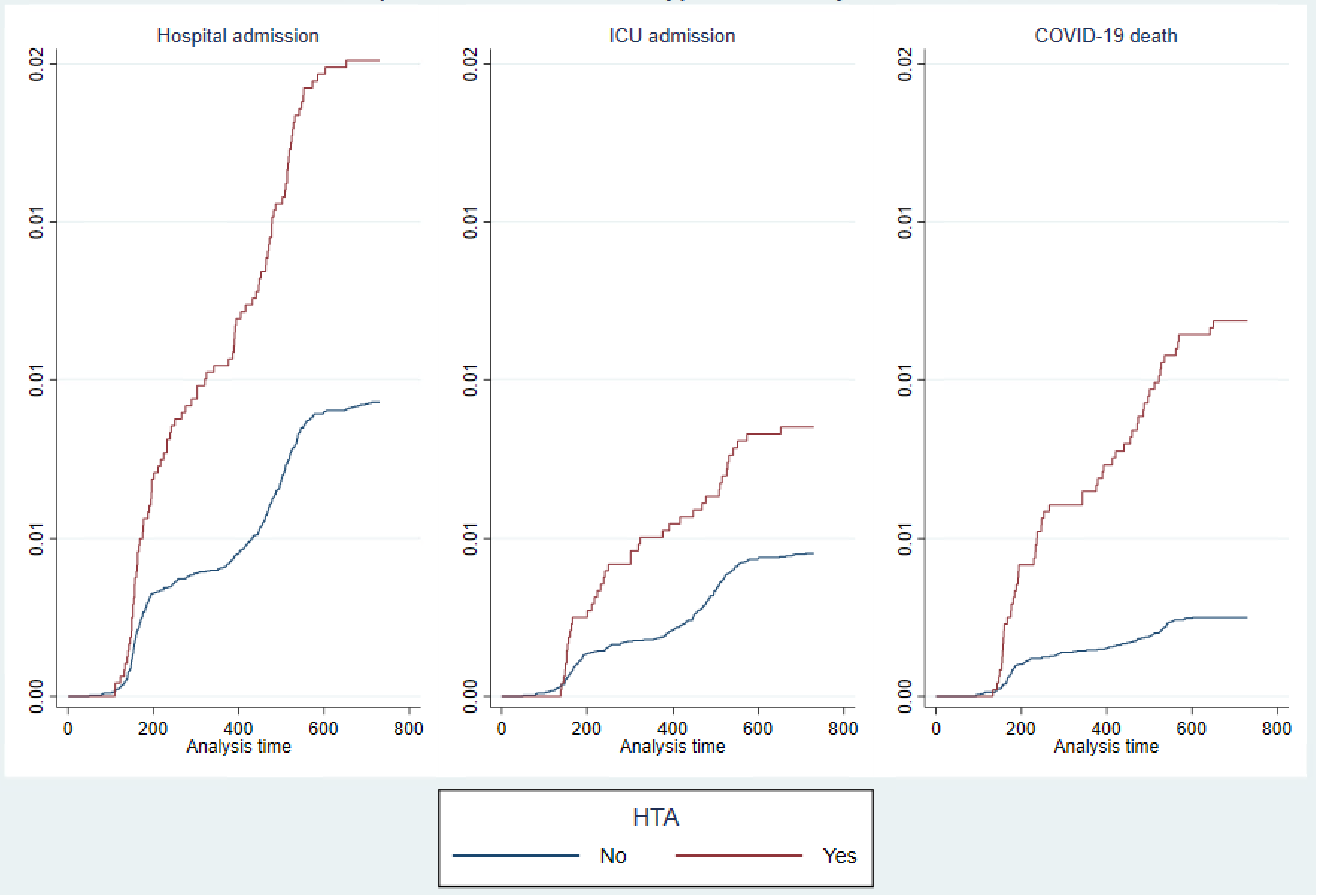


1. **Diabetes Mellitus**


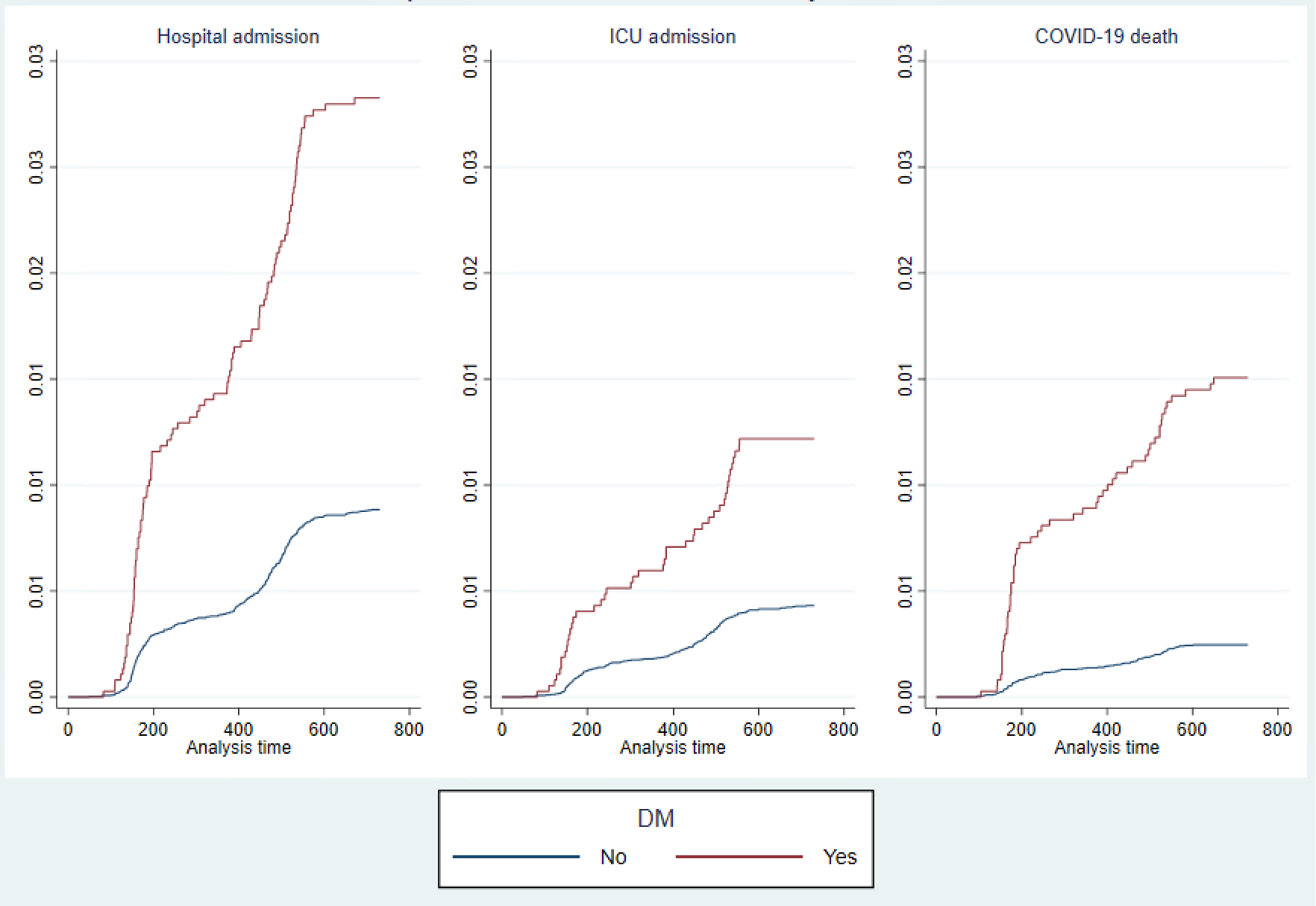


1. **Depression**


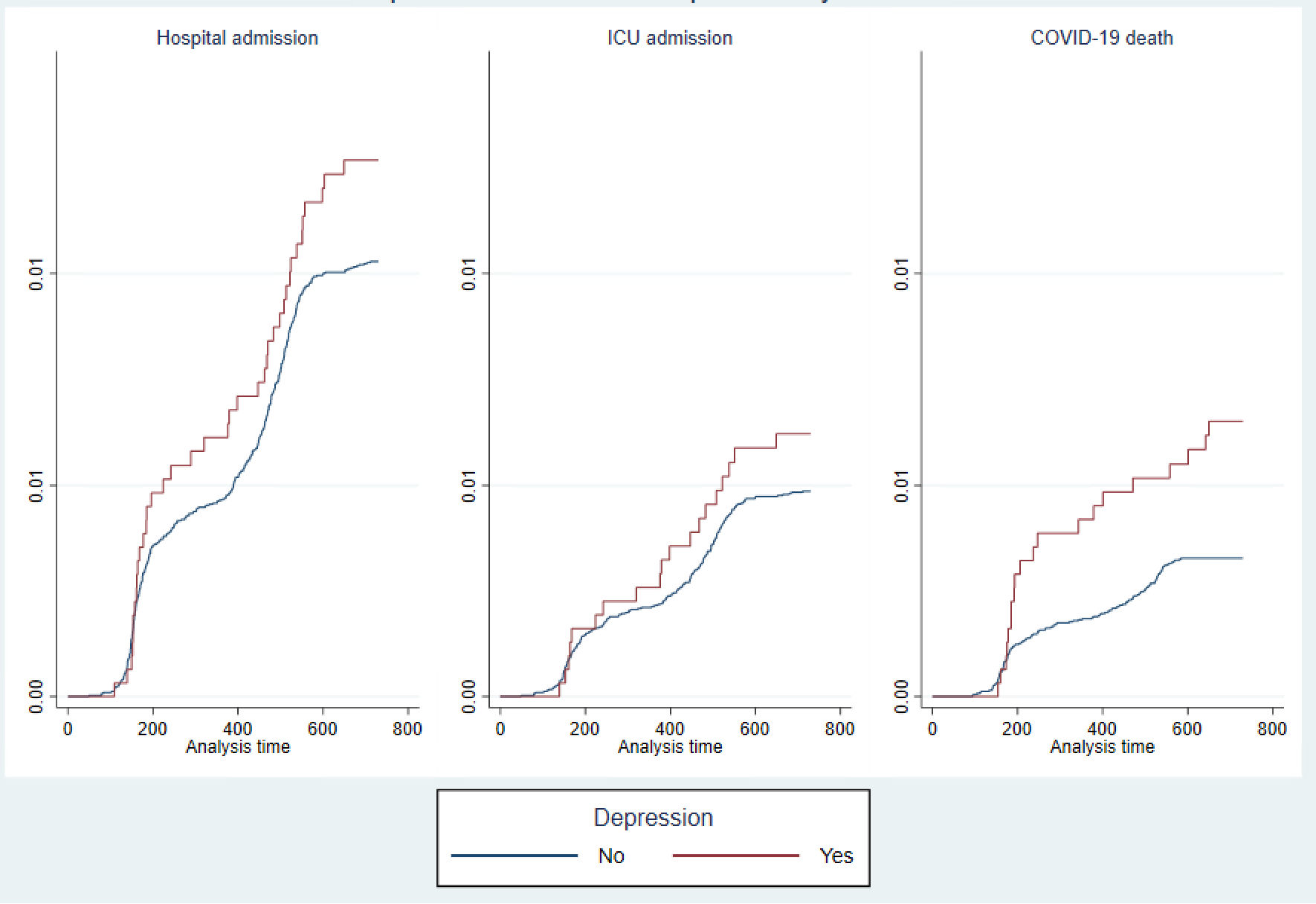


1. **Influenza vaccine**

**
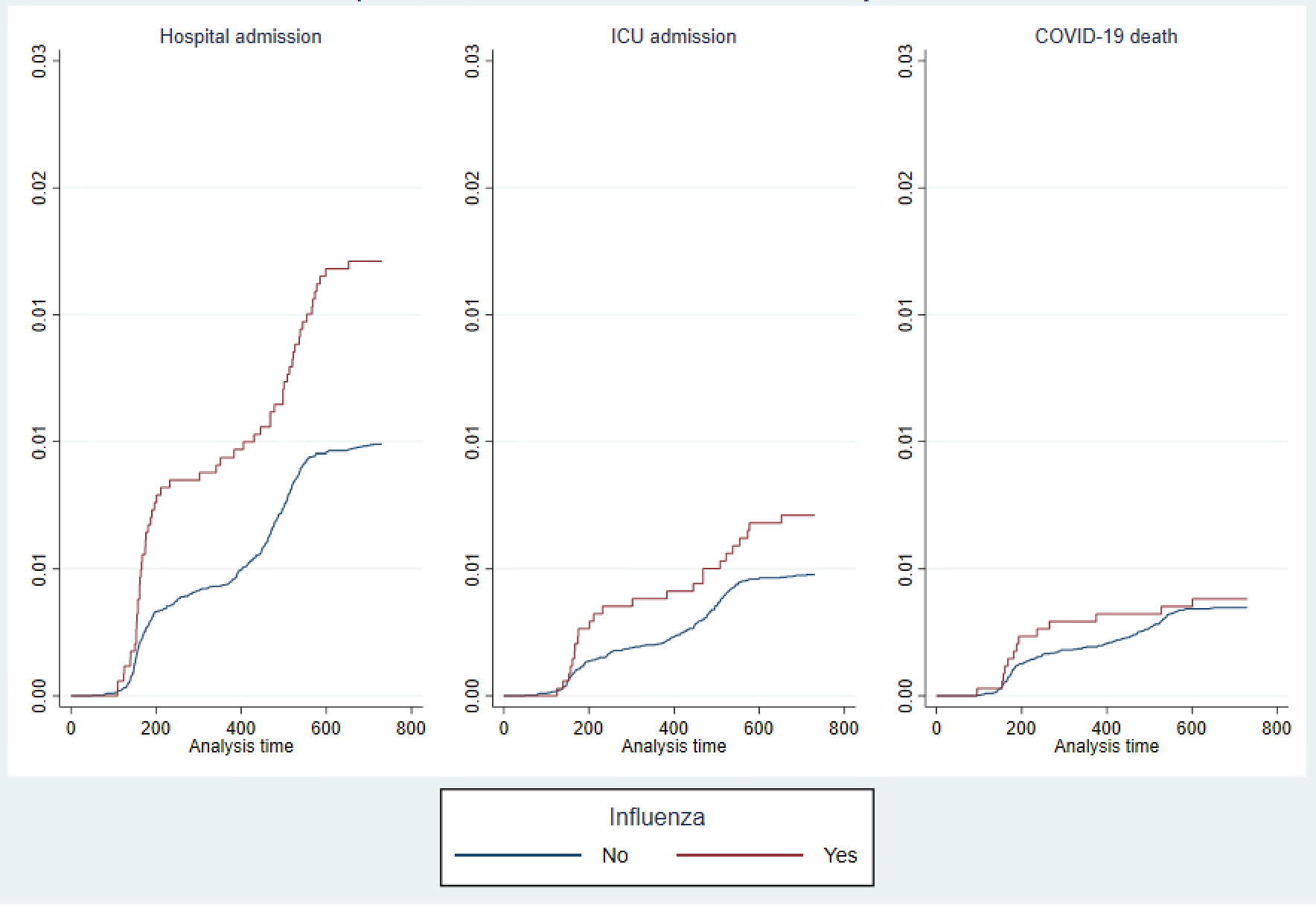
**

1. **Covid-19 vaccine**


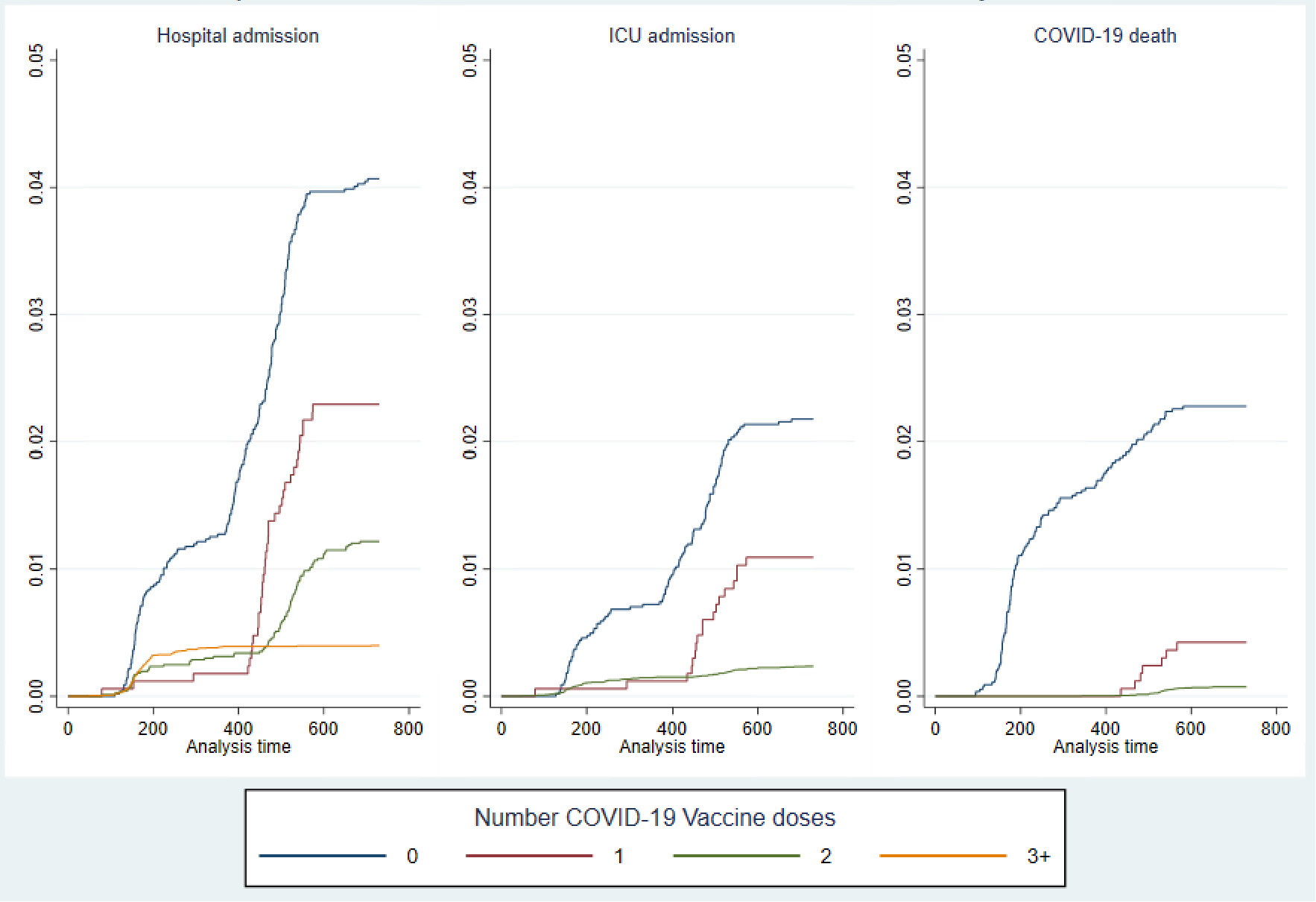

Supplement: S2 Fig — (DOCX) [file pone.0314376.s002.docx]
