# Supplementary material for: Association between demographic, clinical characteristics and severe complications by SARS-CoV-2 infection in a community-based healthcare network in Chile
Source: PLoS One. 2024 Dec 30;19(12):e0314376. doi: 10.1371/journal.pone.0314376 (PMC11684639; doi:10.1371/journal.pone.0314376)
Supplement: S1 Table — (DOCX) [file pone.0314376.s003.docx]

|  | Chile | Metropolitan Region | Ancora network |
| --- | --- | --- | --- |
| Age (mean) | 35.8 | 35.6 | 33.1 |
| <15 years old | 20.1% | 19.4% | 24.5% |
| >65 years old | 11.4% | 10.8% | 8.2% |
| % female | 51.1% | 51.3% | 53.9% |
| FONASA categories |  |  |  |
| A | 19.6% | 18.4% | 18.2% |
| B | 39.4% | 37.8% | 33.9% |
| C | 16.3% | 17.2% | 16.7% |
| D | 24.7% | 26.6% | 25.5% |
