# Supplementary material for: Association between demographic, clinical characteristics and severe complications by SARS-CoV-2 infection in a community-based healthcare network in Chile
Source: PLoS One. 2024 Dec 30;19(12):e0314376. doi: 10.1371/journal.pone.0314376 (PMC11684639; doi:10.1371/journal.pone.0314376)
Supplement: S2 Table — (DOCX) [file pone.0314376.s004.docx]

**S3 Table. Codes to define comorbidities**

program drop comorbilidad

program define comorbilidad

*HTA (1)*

gen HTA=1 if strmatch(`1', "*HTA*")

replace HTA=1 if strmatch(`1', "*hiperten*")

replace HTA=1 if strmatch(`1', "*HIPERTEN*")

replace HTA=. if strmatch(`1', "*SIN HTA*")

replace HTA=. if strmatch(`1', "*SOSPECHA*")

replace HTA=. if strmatch(`1', "*SOSP.*")

replace HTA=. if strmatch(`1', "*MIEDO*")

*DM (2)*

gen DM=1 if strmatch(`1', "*DM*")

replace DM=1 if strmatch(`1', "*diabet*")

replace DM=1 if strmatch(`1', "*DIABETE*")

replace DM=. if strmatch(`1', "*SOSPECHA*")

replace DM=. if strmatch(`1', "*SOSP.*")

replace DM=. if strmatch(`1', "*MIEDO*")

replace DM=. if strmatch(`1', "*NO DIABETES*")

replace DM=. if strmatch(`1', "*PRE DIAB*")

replace DM=. if strmatch(`1', "*PREDIAB*")

replace DM=. if strmatch(`1', "*NO DM*")

replace DM=. if strmatch(`1', "*TDM*")

*Depresion (3)*

gen depre=1 if strmatch(`1', "Depres*")

replace depre=1 if strmatch(`1', "*DEPRES*")

replace depre=1 if strmatch(`1', "*ANIMO*")

*Asma (4)*

gen asma=1 if strmatch(`1', "*asma*")

replace asma=1 if strmatch(`1', "*ASMA*")

replace asma=. if strmatch(`1', "*LACTANTE*")

replace asma=. if strmatch(`1', "*UREAPLASMA*")

replace asma=. if strmatch(`1', "*FANTASMA*")

replace asma=. if strmatch(`1', "*XANTELASMA*")

replace asma=. if strmatch(`1', "*SOSPECHA*")

replace asma=. if strmatch(`1', "*SOSP*")

replace asma=. if strmatch(`1', "*OBS ASMA*")

replace asma=. if strmatch(`1', "*MELASMA*")

*Demencia*

gen demencia=1 if strmatch(`1', "*DEMENCIA*")

replace demencia=. if strmatch(`1', "*SOSPECHA*")

replace demencia=. if strmatch(`1', "*MIEDO*")

replace demencia=1 if strmatch(`1', "*ALZHEIM*")

replace demencia=1 if strmatch(`1', "*ALZAIM*")

*COPD*

gen COPD=1 if strmatch(`1', "*COPD*")

replace COPD=1 if strmatch(`1', "*EPOC*")

replace COPD=1 if strmatch(`1', "*EBOC*")

replace COPD=1 if strmatch(`1', "*ENFISEMA*")

replace COPD=1 if strmatch(`1', "*BRONQUITIS CRONICA*")

replace COPD=1 if strmatch(`1', "*LCFA*")

replace COPD=. if strmatch(`1', "*SOSPECHA*")

replace COPD=. if strmatch(`1', "*OBS EPOC*")

*Cardiopatia coronaria*

gen coronaria=1 if strmatch(`1', "*CORONARIA*")

replace coronaria=1 if strmatch(`1', "*IAM*")

replace coronaria=1 if strmatch(`1', "*BYPASS*")

replace coronaria=1 if strmatch(`1', "*ANGINA*")

replace coronaria=. if strmatch(`1', "*ABSCESO*")

replace coronaria=. if strmatch(`1', "*HERPANGINA*")

replace coronaria=. if strmatch(`1', "*WILLIAM*")

replace coronaria=. if strmatch(`1', "*GASTRICO*")

replace coronaria=1 if `2'=="K76" | `2'=="K75" | `2'=="K74"

replace coronaria=. if strmatch(`1', "*ANSIEDAD*")

*AVE*

gen cerebrovascular=1 if strmatch(`1', "*ACCIDENTE VASCULAR*")

replace cerebrovascular=1 if `2'=="K89" | `2'=="K90" | `2'=="K91"

replace cerebrovascular=1 if strmatch(`1', "*AVE*")

replace cerebrovascular=1 if strmatch(`1', "*ACV*")

replace cerebrovascular=. if strmatch(`1', "*CEFALEA / MIGRAÑA*")

replace cerebrovascular=. if strmatch(`1', "*APNEA*")

replace cerebrovascular=. if strmatch(`1', "*GRAVE*")

replace cerebrovascular=. if strmatch(`1', "*CAVERNOSO*")

replace cerebrovascular=. if strmatch(`1', "*SUPRAVENTRICULAR*")

replace cerebrovascular=. if strmatch(`1', "*CAVERNOMA*")

replace cerebrovascular=. if strmatch(`1', "*EPILEPSIA*")

*CANCER*

gen cancer=1 if strmatch(`1', "*CANCER*")

replace cancer=1 if strmatch(`1', "*NEOPL*")

replace cancer=1 if strmatch(`1', "*MALIGN*")

replace cancer=1 if strmatch(`1', "*LINFOMA*")

replace cancer=1 if strmatch(`1', "*LEUCEMIA*")

replace cancer=1 if strmatch(`1', "*MIELOMA*")

replace cancer=1 if strmatch(`1', "*MELANOMA*")

replace cancer=. if strmatch(`1', "*MIEDO*")

replace cancer=. if strmatch(`1', "*SOSPECHA*")

replace cancer=. if strmatch(`1', "*BENIGN*")

*EPI*

gen EPI=1 if strmatch(`1', "*EPILE*")

replace EPI=1 if strmatch(`1', "*CONVULS*")

*DAÑO RENAL*

gen RENAL=1 if strmatch(`1', "*RENAL*")

replace RENAL=. if strmatch(`1', "*COLICO*")

replace RENAL=. if strmatch(`1', "*LITIASIS*")

replace RENAL=. if strmatch(`1', "*PIELO*")

replace RENAL=. if strmatch(`1', "*CALCULO*")

replace RENAL=1 if strmatch(`1', "*DIALISIS*")

replace RENAL=. if strmatch(`1', "*QUISTE*")

replace RENAL=. if strmatch(`1', "*MASA*")

replace RENAL=. if strmatch(`1', "*SUPRAR*")

replace RENAL=. if strmatch(`1', "*NEO*")

replace RENAL=. if strmatch(`1', "*NODULO*")

replace RENAL=. if strmatch(`1', "*LESION*")

replace RENAL=. if strmatch(`1', "*MIEDO*")

replace RENAL=. if strmatch(`1', "*MONOR*")

replace RENAL=. if strmatch(`1', "*ECOGRAF*")

*DAÑO HEPATICO*

gen HEPATICO=1 if strmatch(`1', "*HEPATIC*")

replace HEPATICO=1 if strmatch(`1', "*CIRROSIS*")

replace HEPATICO=. if strmatch(`1', "*PRUEBAS HEPATICAS*")

replace HEPATICO=. if strmatch(`1', "*ESTEATOSIS*")

replace HEPATICO=. if strmatch(`1', "*ABSESO*")

replace HEPATICO=1 if strmatch(`1', "*VARICES ESOFAGICAS*")

replace HEPATICO=1 if strmatch(`1', "*ACITIS*")

replace HEPATICO=1 if strmatch(`1', "*CHILD*")

replace HEPATICO=1 if strmatch(`1', "*DHC*")

replace HEPATICO=. if strmatch(`1', "*QUISTE*")

replace HEPATICO=. if strmatch(`1', "*COLESTASIA*")

replace HEPATICO=. if strmatch(`1', "*LESION*")

replace HEPATICO=. if strmatch(`1', "*QUISTE*")

replace HEPATICO=. if strmatch(`1', "*ALTERAC*")

replace HEPATICO=. if strmatch(`1', "*ABSCESO*")

replace HEPATICO=. if strmatch(`1', "*NEOPLASIA*")

replace HEPATICO=. if strmatch(`1', "*HIPERPLASIA NOCULAR*")

replace HEPATICO=. if strmatch(`1', "*HEMANGIOMA*")

*ENF PEPTICA*

gen UG=1 if strmatch(`1', "*HEMORRAGIA*")

replace UG=1 if strmatch(`1', "*PEPTIC*")

replace UG=1 if strmatch(`1', "*DUODE*")

replace UG=1 if strmatch(`1', "*ULCERA*")

replace UG=. if strmatch(`1', "*MIEDO*")

replace UG=. if strmatch(`1', "*SUBARACNOIDEA*")

replace UG=. if strmatch(`1', "*PRES*")

replace UG=1 if strmatch(`1', "*GASTRIC*")

replace UG=. if strmatch(`1', "*VARICOS*")

replace UG=. if strmatch(`1', "*PIE*")

replace UG=. if strmatch(`1', "*VENOS*")

replace UG=. if strmatch(`1', "*GANGLIO*")

replace UG=. if strmatch(`1', "*VULVA*")

replace UG=. if strmatch(`1', "*SACRA*")

replace UG=. if strmatch(`1', "*BOCA*")

replace UG=. if strmatch(`1', "*AFTA*")

replace UG=. if strmatch(`1', "*DECUBITO*")

replace UG=. if strmatch(`1', "*DUODENITIS*")

replace UG=. if strmatch(`1', "*CORNEA*")

replace UG=. if strmatch(`1', "*EEII*")

replace UG=. if strmatch(`1', "*TOBILLO*")

replace UG=. if strmatch(`1', "*DIABET*")

replace UG=. if strmatch(`1', "*BYPASS*")

replace UG=. if strmatch(`1', "*SUBCONJ*")

replace UG=. if strmatch(`1', "*CANCER*")

replace UG=. if strmatch(`1', "*DOLOR*")

replace UG=. if strmatch(`1', "*NASAL*")

replace UG=. if strmatch(`1', "*ENF VIRAL GASTRICA*")

replace UG=. if strmatch(`1', "*HIPEREMESIS/*")

replace UG=. if strmatch(`1', "*POSTMENOPAUSICA*")

replace UG=. if strmatch(`1', "*INFERIOR*")

replace UG=. if strmatch(`1', "*ENCIAS*")

replace UG=. if strmatch(`1', "*PLANTAR*")

replace UG=. if strmatch(`1', "*GLUTEO*")

replace UG=. if strmatch(`1', "*SUBDURAL*")

replace UG=. if strmatch(`1', "*RETINAL*")

replace UG=. if strmatch(`1', "*PENE*")

replace UG=. if strmatch(`1', "*HERIDA*")

replace UG=. if strmatch(`1', "*NEVUS*")

replace UG=. if strmatch(`1', "*ANEMIA*")

*HIPOTIROIDISMO*

gen HIPO=1 if strmatch(`1', "*HIPOT*")

replace HIPO=. if strmatch( `1', "*HIPOFISIARIA*")

replace HIPO=. if strmatch( `1', "*HIPOCONDRIA*")

replace HIPO=. if strmatch( `1', "*HIPOTENSION*")

foreach var of varlist HTA-HIPO{

replace `var'=0 if `var'==.

}

end
