# Supplementary material for: Association between demographic, clinical characteristics and severe complications by SARS-CoV-2 infection in a community-based healthcare network in Chile
Source: PLoS One. 2024 Dec 30;19(12):e0314376. doi: 10.1371/journal.pone.0314376 (PMC11684639; doi:10.1371/journal.pone.0314376)
Supplement: S3 Table — (DOCX) [file pone.0314376.s005.docx]

S5 table. Results from the test of proportional hazards assumption (phtest)

1. **All cohort**

|  | **A hospital admission for COVID infection** | | | | **ICU admission for COVID infection** | | | | **Death due to COVID infection** | | | |
| --- | --- | --- | --- | --- | --- | --- | --- | --- | --- | --- | --- | --- |
|  | **rho** | **chi2** | **df** | **Prob>chi2** | **rho** | **chi2** | **df** | **Prob>chi2** | **rho** | **chi2** | **df** | **Prob>chi2** |
| Age (Ref 18 to 34) |  |  |  |  |  |  |  |  |  |  |  |  |
| 35 to 54 | -0.003 | 0.01 | 1 | 0.942 | -0.080 | 1.34 | 1 | 0.246 |  |  |  |  |
| 55 to 69 | -0.050 | 1.16 | 1 | 0.282 | -0.186 | 7.69 | 1 | 0.006 | -0.039 | 0.25 | 1 | 0.618 |
| >70 | -0.018 | 0.15 | 1 | 0.697 | -0.173 | 6.7 | 1 | 0.010 | 0.025 | 0.11 | 1 | 0.741 |
| Sex (Ref Male) |  |  |  |  |  |  |  |  |  |  |  |  |
| Female | 0.170 | 13.43 | 1 | <0.001 | 0.188 | 7.92 | 1 | 0.005 | 0.120 | 1.87 | 1 | 0.171 |
| Fonasa (Ref A, lowest income) |  |  |  |  |  |  |  |  |  |  |  |  |
| B | -0.027 | 0.32 | 1 | 0.571 | -0.032 | 0.22 | 1 | 0.640 | -0.040 | 0.21 | 1 | 0.645 |
| C | 0.008 | 0.03 | 1 | 0.856 | -0.065 | 0.91 | 1 | 0.341 | -0.089 | 1.25 | 1 | 0.264 |
| D (highest income) | -0.027 | 0.33 | 1 | 0.566 | -0.056 | 0.66 | 1 | 0.417 | -0.064 | 0.63 | 1 | 0.428 |
| HTA (Ref No) |  |  |  |  |  |  |  |  |  |  |  |  |
| Yes | -0.006 | 0.02 | 1 | 0.898 | -0.027 | 0.16 | 1 | 0.691 | 0.157 | 3.34 | 1 | 0.068 |
| DM (Ref No) |  |  |  |  |  |  |  |  |  |  |  |  |
| Yes | 0.009 | 0.04 | 1 | 0.838 | -0.039 | 0.32 | 1 | 0.570 | 0.034 | 0.2 | 1 | 0.655 |
| Depression (Ref No) |  |  |  |  |  |  |  |  |  |  |  |  |
| Yes | -0.073 | 2.5 | 1 | 0.114 | -0.045 | 0.46 | 1 | 0.498 | 0.004 | 0 | 1 | 0.955 |
| Frequently dispatched drugs | 0.054 | 1.34 | 1 | 0.247 | 0.109 | 2.3 | 1 | 0.130 | -0.101 | 1.85 | 1 | 0.174 |
| Number of doctors´ contacts (1 year) | 0.033 | 0.38 | 1 | 0.539 | 0.008 | 0.01 | 1 | 0.922 | 0.064 | 0.54 | 1 | 0.461 |
| Number of nurses´ contacts (1 year) | 0.012 | 0.06 | 1 | 0.800 | 0.183 | 3.31 | 1 | 0.069 | 0.162 | 3.67 | 1 | 0.055 |
| Number of Covid-19 vaccine doses (Ref 0) |  |  |  |  |  |  |  |  |  |  |  |  |
| 1 | 0.074 | 2.48 | 1 | 0.116 |  |  |  |  |  |  |  |  |
| 2 | -0.010 | 0.04 | 1 | 0.838 | 0.094 | 1.9 | 1 | 0.169 | 0.300 | 13.17 | 1 | <0.001 |
| 3+ | -0.557 | 187.18 | 1 | <0.001 | -0.203 | 9.43 | 1 | 0.002 | 0.573 | 51.2 | 1 | <0.001 |
| Influenza vaccine previous year (Ref No) |  |  |  |  |  |  |  |  |  |  |  |  |
| Yes | 0.153 | 11.9 | 1 | 0.001 | 0.029 | 0.2 | 1 | 0.659 | -0.282 | 13.4 | 1 | <0.001 |
|  |  |  |  |  |  |  |  |  |  |  |  |  |
| Global test |  | 257.38 | 18 | <0.001 |  | 46.57 | 16 | <0.001 |  | 80.38 | 15 | <0.001 |

1. **Subgroup analysis**

**b.1. A hospital admission due to COVID infection**

|  | **A hospital admission for COVID infection** | | | | | | | |
| --- | --- | --- | --- | --- | --- | --- | --- | --- |
|  | **Male** | | | | **Female** | | | |
|  | **rho** | **chi2** | **df** | **Prob>chi2** | **rho** | **chi2** | **df** | **Prob>chi2** |
|  |  |  |  |  |  |  |  |  |
| Age (Ref 18 to 34) |  |  |  |  |  |  |  |  |
| 35 to 54 | 0.023 | 0.12 | 1 | 0.727 | -0.035 | 0.23 | 1 | 0.628 |
| 55 to 69 | -0.038 | 0.33 | 1 | 0.568 | -0.086 | 1.69 | 1 | 0.194 |
| >70 | 0.000 | 0 | 1 | 0.999 | -0.055 | 0.66 | 1 | 0.415 |
| Fonasa (Ref A, lowest income) |  |  |  |  |  |  |  |  |
| B | -0.054 | 0.67 | 1 | 0.415 | 0.037 | 0.29 | 1 | 0.589 |
| C | -0.009 | 0.02 | 1 | 0.892 | 0.013 | 0.04 | 1 | 0.850 |
| D (highest income) | -0.062 | 0.89 | 1 | 0.345 | 0.014 | 0.05 | 1 | 0.828 |
| HTA (Ref No) |  |  |  |  |  |  |  |  |
| Yes | 0.060 | 0.9 | 1 | 0.342 | -0.058 | 0.82 | 1 | 0.367 |
| DM (Ref No) |  |  |  |  |  |  |  |  |
| Yes | 0.052 | 0.68 | 1 | 0.408 | -0.023 | 0.14 | 1 | 0.704 |
| Depression (Ref No) |  |  |  |  |  |  |  |  |
| Yes | -0.037 | 0.33 | 1 | 0.564 | -0.102 | 2.45 | 1 | 0.117 |
| Frequently dispatched drugs | -0.033 | 0.28 | 1 | 0.594 | 0.165 | 6.74 | 1 | 0.009 |
| Number of doctors´ contacts | 0.037 | 0.25 | 1 | 0.619 | 0.016 | 0.04 | 1 | 0.841 |
| Number of nurses´ contacts | 0.123 | 3.28 | 1 | 0.070 | -0.086 | 1.08 | 1 | 0.298 |
| Number of Covid-19 vaccine doses (Ref 0) |  |  |  |  |  |  |  |  |
| 1 | 0.167 | 6.39 | 1 | 0.012 | 0.034 | 0.28 | 1 | 0.596 |
| 2 | 0.105 | 2.48 | 1 | 0.115 | 0.044 | 0.45 | 1 | 0.500 |
| 3+ | -0.463 | 55.35 | 1 | <0.001 | -0.527 | 73.21 | 1 | <0.001 |
| Influenza vaccine previous year (Ref No) |  |  |  |  |  |  |  |  |
| Yes | 0.049 | 0.6 | 1 | 0.440 | 0.146 | 5.15 | 1 | 0.023 |
| Pregnant or puerperal women (Ref No) |  |  |  |  | 0.014 | 0.04 | 1 | 0.841 |
| Yes |  |  |  |  |  |  |  |  |
| Global test |  | 109.2 | 16 | <0.001 |  | 106.91 | 17 | <0.001 |

**b.2. ICU admission due to COVID infection**

|  | **ICU admission due to COVID infection** | | | | | | | |
| --- | --- | --- | --- | --- | --- | --- | --- | --- |
|  | **Male** | | | | **Female** | | | |
|  | **rho** | **chi2** | **df** | **Prob>chi2** | **rho** | **chi2** | **df** | **Prob>chi2** |
|  |  |  |  |  |  |  |  |  |
| Age (Ref 18 to 34) |  |  |  |  |  |  |  |  |
| 35 to 54 | -0.040 | 0.18 | 1 | 0.669 | -0.083 | 0.57 | 1 | 0.449 |
| 55 to 69 | -0.130 | 1.94 | 1 | 0.163 | -0.188 | 3.51 | 1 | 0.061 |
| >70 | -0.143 | 2.48 | 1 | 0.115 | -0.086 | 0.66 | 1 | 0.418 |
| Fonasa (Ref A, lowest income) |  |  |  |  |  |  |  |  |
| B | 0.025 | 0.07 | 1 | 0.789 | -0.053 | 0.26 | 1 | 0.613 |
| C | -0.039 | 0.18 | 1 | 0.668 | -0.041 | 0.17 | 1 | 0.681 |
| D (highest income) | -0.062 | 0.45 | 1 | 0.502 | -0.012 | 0.02 | 1 | 0.900 |
| HTA (Ref No) |  |  |  |  |  |  |  |  |
| Yes | 0.074 | 0.72 | 1 | 0.397 | -0.131 | 1.65 | 1 | 0.200 |
| DM (Ref No) |  |  |  |  |  |  |  |  |
| Yes | -0.013 | 0.02 | 1 | 0.899 | -0.117 | 1.41 | 1 | 0.235 |
| Depression (Ref No) |  |  |  |  |  |  |  |  |
| Yes | -0.017 | 0.04 | 1 | 0.850 | -0.066 | 0.44 | 1 | 0.506 |
| Frequently dispatched drugs | -0.018 | 0.04 | 1 | 0.846 | 0.275 | 6.19 | 1 | 0.013 |
| Number of doctors´ contacts | 0.008 | 0.01 | 1 | 0.942 | 0.042 | 0.13 | 1 | 0.714 |
| Number of nurses´ contacts | 0.174 | 1.94 | 1 | 0.163 | 0.152 | 0.85 | 1 | 0.356 |
| Number of Covid-19 vaccine doses (Ref 0) |  |  |  |  |  |  |  |  |
| 1 | 0.166 | 3.17 | 1 | 0.075 | -0.017 | 0.03 | 1 | 0.865 |
| 2 | -0.060 | 0.43 | 1 | 0.511 | -0.372 | 14.07 | 1 | <0.001 |
| Influenza vaccine previous year (Ref No) |  |  |  |  |  |  |  |  |
| Yes | -0.048 | 0.29 | 1 | 0.589 | -0.011 | 0.01 | 1 | 0.913 |
| Pregnant or puerperal women (Ref No) |  |  |  |  |  |  |  |  |
| Yes |  |  |  |  | 0.152 | 2.34 | 1 | 0.126 |
| **Global test** |  | 16.88 | 15 | 0.326 |  | 28.77 | 16 | 0.026 |

**b.3. Death due to COVID infection**

|  | **ICU admissions** | | | | | | | |
| --- | --- | --- | --- | --- | --- | --- | --- | --- |
|  | **Male** | | | | **Female** | | | |
|  | **rho** | **chi2** | **df** | **Prob>chi2** | **rho** | **chi2** | **df** | **Prob>chi2** |
|  |  |  |  |  |  |  |  |  |
| Age (Ref 18 to 54) |  |  |  |  |  |  |  |  |
| 55 to 69 | -0.036 | 0.11 | 1 | 0.745 | 0.085 | 0.6 | 1 | 0.437 |
| >70 | -0.002 | 0 | 1 | 0.989 | 0.136 | 1.51 | 1 | 0.219 |
| Fonasa (Ref A, lowest income) |  |  |  |  |  |  |  |  |
| B | 0.018 | 0.02 | 1 | 0.886 | -0.084 | 0.54 | 1 | 0.462 |
| C | -0.029 | 0.06 | 1 | 0.813 | -0.124 | 1.28 | 1 | 0.259 |
| D (highest income) | -0.067 | 0.3 | 1 | 0.585 | -0.071 | 0.42 | 1 | 0.519 |
| HTA (Ref No) |  |  |  |  |  |  |  |  |
| Yes | 0.084 | 0.42 | 1 | 0.518 | 0.204 | 3.51 | 1 | 0.061 |
| DM (Ref No) |  |  |  |  |  |  |  |  |
| Yes | 0.069 | 0.38 | 1 | 0.540 | -0.096 | 0.86 | 1 | 0.353 |
| Depression (Ref No) |  |  |  |  |  |  |  |  |
| Yes | -0.023 | 0.04 | 1 | 0.834 | 0.105 | 0.94 | 1 | 0.332 |
| Frequently dispatched drugs | -0.108 | 1.03 | 1 | 0.311 | -0.085 | 0.63 | 1 | 0.429 |
| Number of doctors´ contacts | 0.019 | 0.02 | 1 | 0.881 | 0.100 | 0.47 | 1 | 0.495 |
| Number of nurses´ contacts | 0.036 | 0.05 | 1 | 0.815 | 0.253 | 5.11 | 1 | 0.024 |
| Number of Covid-19 vaccine doses (Ref 0) |  |  |  |  |  |  |  |  |
| 1 | 0.178 | 2.14 | 1 | 0.143 | 0.421 | 14.16 | 1 | <0.001 |
| 2 | 0.584 | 25.38 | 1 | <0.001 | 0.496 | 20.04 | 1 | <0.001 |
| Influenza vaccine previous year (Ref No) |  |  |  |  |  |  |  |  |
| Yes | -0.285 | 5.88 | 1 | 0.015 | -0.209 | 3.61 | 1 | 0.058 |
| **Global test** |  | 32.75 | 14 | 0.003 |  | 45.91 | 14 | <0.001 |
