# Supplementary material for: Association between demographic, clinical characteristics and severe complications by SARS-CoV-2 infection in a community-based healthcare network in Chile
Source: PLoS One. 2024 Dec 30;19(12):e0314376. doi: 10.1371/journal.pone.0314376 (PMC11684639; doi:10.1371/journal.pone.0314376)
Supplement: S4 Table — (DOCX) [file pone.0314376.s006.docx]

|  | **Healthcare center** | | | | | | |
| --- | --- | --- | --- | --- | --- | --- | --- |
|  | **Center 1** | | **Center 2** | | **Center 3** | |  |
|  | n= | 12,192 | n= | 15,996 | n= | 16,486 |  |
|  | No. | % | No. | % | No. | % | p-value |
| Days of follow-up |  |  |  |  |  |  |  |
| Mean (sd) | 42.62 | (16.54) | 45.78 | (18.12) | 44.10 | (16.94) | <0.001 |
| Age |  |  |  |  |  |  |  |
| Mean (sd) | 706.65 | (97.25) | 712.42 | (85.94) | 717.98 | (70.91) | <0.001 |
| Age categories (Ref 18 to 34) |  |  |  |  |  |  |  |
| 35 to 54 | 4,174 | 34.20% | 5,599 | 35.00% | 5,740 | 34.80% | <0.001 |
| 55 to 69 | 2,514 | 20.60% | 3,100 | 19.40% | 3,543 | 21.50% |  |
| >70 | 727 | 6.00% | 1,855 | 11.60% | 1,200 | 7.30% |  |
| Sex (Ref Male) |  |  |  |  |  |  |  |
| Female | 6,320 | 51.80% | 8,915 | 55.70% | 9,620 | 58.40% | <0.001 |
| Tramo fonasa (Ref A lowest income) |  |  |  |  |  |  |  |
| B | 3,841 | 31.50% | 5,679 | 35.50% | 5,706 | 34.60% | <0.001 |
| C | 1,856 | 15.20% | 2,775 | 17.30% | 2,883 | 17.50% |  |
| D (highest income) | 2,776 | 22.80% | 4,716 | 29.50% | 5,249 | 31.80% |  |
| Missing | 571 | 4.70% | 692 | 4.30% | 819 | 5.00% |  |
| Count of comorbidities (Ref 0) |  |  |  |  |  |  |  |
| 1 to 2 | 3,033 | 24.90% | 4,419 | 27.60% | 4,087 | 24.80% | <0.001 |
| >=3 | 229 | 1.90% | 483 | 3.00% | 338 | 2.10% |  |
| Frequently dispatched drugs (Ref 0) |  |  |  |  |  |  |  |
| 1 to 2 | 650 | 5.30% | 1,276 | 8.00% | 1,477 | 9.00% | <0.001 |
| 3 to 5 | 1,005 | 8.20% | 1,689 | 10.60% | 1,623 | 9.80% |  |
| >=6 | 1,433 | 11.80% | 1,614 | 10.10% | 1,177 | 7.10% |  |
| Record of Comorbidities in Primary Care |  |  |  |  |  |  |  |
| HTA (Ref No) | 1,074 | 8.80% | 2,237 | 14.00% | 1,887 | 11.40% | <0.001 |
| DM (Ref No) | 1,228 | 10.10% | 1,578 | 9.90% | 1,375 | 8.30% | <0.001 |
| Depression (Ref No) | 979 | 8.00% | 1,403 | 8.80% | 1,124 | 6.80% | <0.001 |
| Mean (sd) number of encounters with |  |  |  |  |  |  |  |
| Physicians | 0.01 | (0.13) | 0.01 | (0.13) | 0.03 | (0.19) | <0.001 |
| Nurses | 0.18 | (0.86) | 0.18 | (0.71) | 0.14 | (0.71) | <0.001 |
| Influenza vaccine previous year (Ref No) | 701 | 5.70% | 1,529 | 9.60% | 1,191 | 7.20% | <0.001 |
| Number of Covid vaccine doses received (Ref 0) |  |  |  |  |  |  |  |
| 1 | 739 | 6.10% | 491 | 3.10% | 465 | 2.80% | <0.001 |
| 2 | 2,925 | 24.00% | 2,451 | 15.30% | 2,326 | 14.10% |  |
| 3 | 6,666 | 54.70% | 11,163 | 69.80% | 11,866 | 72.00% |  |
|  |  |  |  |  |  |  |  |
| Pregnant or puerperal women (Ref No) | 158 | 1.30% | 159 | 1.00% | 164 | 1.00% | 0.023 |
|  |  |  |  |  |  |  |  |
| Covid positive (Ref No) | 2,181 | 17.90% | 2,125 | 13.30% | 1,879 | 11.40% | <0.001 |
| Covid hospital admission (Ref No) | 151 | 1.20% | 154 | 1.00% | 150 | 0.90% | 0.016 |
| Covid-ICU hospital admission (Ref No) | 67 | 0.50% | 78 | 0.50% | 71 | 0.40% | 0.356 |
| Covid death (Ref No) | 50 | 0.40% | 52 | 0.30% | 46 | 0.30% | 0.159 |

**S6. Table. Sociodemographic and clinical characteristics by healthcare centre**
