# Supplementary material for: Association between demographic, clinical characteristics and severe complications by SARS-CoV-2 infection in a community-based healthcare network in Chile
Source: PLoS One. 2024 Dec 30;19(12):e0314376. doi: 10.1371/journal.pone.0314376 (PMC11684639; doi:10.1371/journal.pone.0314376)
Supplement: S5 Table — (DOCX) [file pone.0314376.s007.docx]

S7 Table. Hazard Ration for subgroup analysis by sex

|  | **A hospital admission due to COVID infection** | | | | **ICU admission due to COVID infection** | | | | **Death due to COVID infection** | | | |
| --- | --- | --- | --- | --- | --- | --- | --- | --- | --- | --- | --- | --- |
|  | Male | | Female | | Male | | Female | | Male | | Female | |
|  | n=18496 | | n=24096 | | n=18496 | | n=24096 | | n=18496 | | n=24096 | |
|  | HR | 95% CI | HR | 95% CI | HR | 95% CI | HR | 95% CI | HR | 95% CI | HR | 95% CI |
| Age (Ref 18 to 34) |  |  |  |  |  |  |  |  |  |  |  |  |
| 35 to 54 | 1.44 | (1.29,1.60) | 1.2 | (1.11,1.29) | 1.4 | (1.21,1.63) | 1.19 | (1.07,1.32) |  |  |  |  |
| 55 to 69 | 1.71 | (1.53,1.90) | 1.33 | (1.22,1.44) | 1.71 | (1.47,1.99) | 1.23 | (1.09,1.39) | 1.93 | (1.66,2.25) | 1.26 | (1.10,1.45) |
| >70 | 1.8 | (1.60,2.03) | 1.37 | (1.26,1.50) | 1.68 | (1.43,1.99) | 1.17 | (1.01,1.37) | 2.27 | (1.94,2.67) | 1.53 | (1.33,1.76) |
| Fonasa (Ref A, lowest income) |  |  |  |  |  |  |  |  |  |  |  |  |
| B | 1.07 | (0.68,1.66) | 1.49 | (1.00,2.24) | 1.18 | (0.63,2.24) | 0.85 | (0.47,1.55) | 0.77 | (0.37,1.58) | 1.43 | (0.63,3.28) |
| C | 1.46 | (0.92,2.33) | 1.38 | (0.82,2.31) | 1.77 | (0.91,3.42) | 1.38 | (0.69,2.77) | 1.33 | (0.61,2.90) | 0.9 | (0.27,3.06) |
| D (highest income) | 1.32 | (0.86,2.04) | 1.39 | (0.88,2.22) | 1.46 | (0.79,2.71) | 1.64 | (0.91,2.99) | 1.05 | (0.51,2.15) | 1.23 | (0.44,3.45) |
| HTA (Ref no) |  |  |  |  |  |  |  |  |  |  |  |  |
| Yes | 1.47 | (1.06,2.04) | 1.3 | (0.91,1.84) | 1.57 | (1.00,2.47) | 1.13 | (0.63,2.00) | 1.24 | (0.72,2.12) | 1.73 | (0.99,3.04) |
| DM (Ref no) |  |  |  |  |  |  |  |  |  |  |  |  |
| Yes | 1.42 | (0.99,2.03) | 1.24 | (0.86,1.80) | 1.12 | (0.67,1.88) | 1.26 | (0.69,2.29) | 1.7 | (0.93,3.08) | 2.78 | (1.55,4.99) |
| Depressio (Ref no) |  |  |  |  |  |  |  |  |  |  |  |  |
| Yes | 0.94 | (0.50,1.75) | 0.99 | (0.67,1.45) | 0.95 | (0.40,2.22) | 1.4 | (0.81,2.41) | 0.99 | (0.35,2.80) | 1.51 | (0.80,2.86) |
| Frequently dispatched drugs | 1.09 | (1.04,1.15) | 1.12 | (1.08,1.16) | 1.11 | (1.04,1.19) | 1.16 | (1.09,1.23) | 1.01 | (0.93,1.09) | 1.06 | (1.00,1.13) |
| Number of doctors´ contacts (1 year) | 1.61 | (0.96,2.69) | 1.12 | (0.63,1.99) | 2.35 | (1.33,4.13) | 0.85 | (0.30,2.43) | 1.2 | (0.25,5.78) | 1.74 | (0.87,3.49) |
| Number of nurses´ contacts (1 year) | 1.05 | (0.96,1.15) | 1.02 | (0.94,1.11) | 0.94 | (0.79,1.12) | 1.08 | (0.95,1.22) | 1.01 | (0.85,1.21) | 1.14 | (1.07,1.22) |
| Influenza vaccine previous year (Ref no) | 1.45 | (0.93,2.26) | 1.63 | (1.06,2.49) | 1.44 | (0.80,2.60) | 0.84 | (0.39,1.83) | 1.08 | (0.92,1.26) | 0.87 | (0.72,1.05) |
| Pregnant or puerperal women (Ref No) |  |  | 2.89 | (1.41,5.89) |  |  | 3.04 | (1.01,9.14) |  |  |  |  |
| Number of Covid vaccine doses received (Ref 0) |  |  |  |  |  |  |  |  |  |  |  |  |
| 1 | 0.94 | (0.86,1.03) | 0.98 | (0.91,1.06) | 0.91 | (0.80,1.04) | 0.93 | (0.82,1.04) | 0.94 | (0.81,1.08) | 0.7 | (0.50,0.97) |
| 2 | 0.8 | (0.75,0.85) | 0.8 | (0.75,0.85) | 0.62 | (0.57,0.66) | 0.57 | (0.53,0.62) | 0.44 | (0.38,0.50) | 0.54 | (0.49,0.59) |
| 3+ | 0.59 | (0.55,0.62) | 0.54 | (0.51,0.58) |  |  |  |  |  |  |  |  |
