# Supplementary material for: Association between demographic, clinical characteristics and severe complications by SARS-CoV-2 infection in a community-based healthcare network in Chile
Source: PLoS One. 2024 Dec 30;19(12):e0314376. doi: 10.1371/journal.pone.0314376 (PMC11684639; doi:10.1371/journal.pone.0314376)
Supplement: S6 Table — (DOCX) [file pone.0314376.s008.docx]

|  |  | | | | |  | | | | |  | | | | |
| --- | --- | --- | --- | --- | --- | --- | --- | --- | --- | --- | --- | --- | --- | --- | --- |
|  | **A hospitalization due to COVID-19 infection** | | | | | **An UCI/UTI hospital admission due to COVID-19 infection** | | | | | **Death due to COVID-19 infection** | | | | |
|  | **n=5759** | | | | | **n=5759** | | | | | **n=5759** | | | | |
|  | HR | | 95% CI | | p-value | HR | | 95% CI | | p-value | HR | | 95% CI | | p-value |
| Age categories (Ref 18 to 34) |  |  | |  | |  |  | |  | |  |  | |  | |
| 35 to 54 | 1.94 | (1.48-2.54) | | <0.001 | | 1.93 | (1.30-2.88) | | 0.001 | |  |  | |  | |
| 55 to 69 | 3.01 | (2.27-3.99) | | <0.001 | | 3.43 | (2.29-5.16) | | <0.001 | | 2.08* | (1.70-2.54) | | <0.001 | |
| >70 | 3.33 | (2.44-4.54) | | <0.001 | | 2.89 | (1.73-4.82) | | <0.001 | | 2.60* | (2.11-3.21) | | <0.001 | |
| Sex (Ref Male) |  |  | |  | |  |  | |  | |  |  | |  | |
| Female | 0.91 | (0.78-1.07) | | 0.267 | | 0.91 | (0.71-1.16) | | 0.456 | | 0.72 | (0.49-1.06) | | 0.095 | |
| Tramo fonasa (Ref A lowest income) |  |  | |  | |  |  | |  | |  |  | |  | |
| B | 1.20 | (0.78-1.84) | | 0.406 | | 0.90 | (0.48-1.68) | | 0.737 | | 0.93 | (0.51-1.68) | | 0.806 | |
| C | 1.13 | (0.68-1.87) | | 0.641 | | 1.35 | (0.68-2.65) | | 0.388 | | 0.68 | (0.33-1.40) | | 0.292 | |
| D (highest income) | 1.13 | (0.72-1.78) | | 0.589 | | 1.25 | (0.67-2.31) | | 0.481 | | 0.74 | (0.38-1.42) | | 0.362 | |
| Record of Comorbidities in Primary Care |  |  | |  | |  |  | |  | |  |  | |  | |
| HTA (Ref No) | 0.98 | (0.68-1.42) | | 0.933 | | 1.30 | (0.77-2.20) | | 0.323 | | 1.20 | (0.79-1.83) | | 0.391 | |
| DM (Ref No) | 1.53 | (1.04-2.25) | | 0.032 | | 0.96 | (0.52-1.79) | | 0.903 | | 2.06 | (1.29-3.26) | | 0.002 | |
| Depression (Ref No) | 0.54 | (0.32-0.91) | | 0.020 | | 0.55 | (0.26-1.16) | | 0.116 | | 1.24 | (0.69-2.23) | | 0.465 | |
| Frequently dispatched drugs | 1.05 | (1.01-1.09) | | 0.019 | | 1.04 | (0.98-1.10) | | 0.227 | | 0.98 | (0.93-1.03) | | 0.404 | |
| Number of encounters with Physicians | 1.50 | (0.89-2.51) | | 0.124 | | 2.19 | (1.18-4.08) | | 0.013 | | 1.76 | (0.89-3.47) | | 0.105 | |
| Number of encounters with nurses | 0.87 | (0.75-1.02) | | 0.080 | | 0.87 | (0.67-1.12) | | 0.279 | | 1.11 | (1.00-1.22) | | 0.040 | |
| Influenza vaccine previous year (Ref No) | 1.27 | 0.95-1.70) | | 0.108 | | 0.90 | (0.41-1.93) | | 0.778 | | 1.02 | (0.81-1.28) | | 0.864 | |
| Number of Covid vaccine doses received (Ref 0) |  |  | |  | |  |  | |  | |  |  | |  | |
| 1 | 0.83 | (0.63-1.10) | | 0.198 | | 0.80 | (0.53-1.22) | | 0.302 | | 0.76** | (0.60-0.96) | | 0.020 | |
| 2 | 0.42 | (0.34-0.53) | | <0.001 | | 0.21 | (0.16-0.28) | | <0.001 | | 0.37** | (0.31-0.43) | | <0.001 | |
| 3 | 0.20 | (0.16-0.25) | | <0.001 | |  |  | |  | |  |  | |  | |

**S8 Table. Hazard Ratio for subgroup analysis for people with a confirmed COVID-19 infection**

HTA: hypertension; DM: diabetes mellitus; (*) Reference 18 to 55 yo; (**) Reference 0, categories 1, 2+ doses.

^1^: age, sex, influenza vaccine and COVID-19 vaccines included as time-varying covariate; ^2^: age, sex and COVID-19 vaccines included as time-varying covariate; ^3^: age, influenza vaccine and COVID-19 vaccines included as time-varying covariate.
