# Supplementary material for: Association between demographic, clinical characteristics and severe complications by SARS-CoV-2 infection in a community-based healthcare network in Chile
Source: PLoS One. 2024 Dec 30;19(12):e0314376. doi: 10.1371/journal.pone.0314376 (PMC11684639; doi:10.1371/journal.pone.0314376)
Supplement: Attachment — Submitted filename: Responses.docx [file pone.0314376.s009.docx]

**Association between demographic, clinical characteristics and severe complications by SARS-CoV-2 infection in a community-based healthcare network in Chile**

**Thank you for the opportunity to respond to the peer reviewers’ comments. We hope we have addressed all the comments. Our responses are included below.**

Journal requirements:

When submitting your revision, we need you to address these additional requirements.

1. Please ensure that your manuscript meets PLOS ONE's style requirements, including those for file naming. The PLOS ONE style templates can be found at

<https://journals.plos.org/plosone/s/file?id=wjVg/PLOSOne_formatting_sample_main_body.pdf> and

<https://journals.plos.org/plosone/s/file?id=ba62/PLOSOne_formatting_sample_title_authors_affiliations.pdf>

**Thank you for highlighting this issue. We made changes to the title page, headings and tables, figures and supporting tables citations across the manuscript to meet PLOS ONE´s style requirements. (Page 1)**

2. In the online submission form, you indicated that your data is available only on request from a third party. Please note that your Data Availability Statement is currently missing [the name of the third party contact or institution / contact details for the third party, such as an email address or a link to where data requests can be made]. Please update your statement with the missing information.

**Thank you for this reminder. We added two paragraphs with the requested information in the Data Availability Statement, with a link to the webpage and contact details of the data controllers as follows: (Page 29, lines 623-631)**

**“The Department of Statistics at the SSMSO holds all the information regarding patients that are enrolled in primary healthcare centres in their area, including administrative and clinical records from primary care and hospital care. The SSMSO act as data controller of the dataset. A formal application process and a data sharing agreement with a Chilean institution is needed in order to access the data. More information about the SSMSO and the Department of Statistics can be found here https://redsalud.ssmso.cl/. Information for data access can be requested at deagis@ssmso.cl.**

**The Unidad de Informatica (UGI) at the Ancora Network manages all clinical and administrative records of the three primary healthcare centres related to the Network. The UGI act as data controller of the dataset. A formal application process and a data sharing agreement with a Chilean institution is needed in order to access the data. More information about the Ancora Network and the UGI can be found here https:// http://www.ancorauc.cl/. Information for data access can be requested at cisauc@ucchristus.cl.”**

3. Your ethics statement should only appear in the Methods section of your manuscript. If your ethics statement is written in any section besides the Methods, please delete it from any other section.

**Thank you. We removed the ethics statement written at the end of the manuscript. (Page 29, lines 615-617)**

4. Please include captions for your Supporting Information files at the end of your manuscript, and update any in-text citations to match accordingly. Please see our Supporting Information guidelines for more information: <http://journals.plos.org/plosone/s/supporting-information>.

**Thank you for raising this comment. We added the Supporting Information’s files separately, amended the in-text citations and included the captions at the end of the manuscript as required. (Page 30, lines 635-642)**

5. Additional Editor Comments:

   Improve the background by updating the literature review, in particular for papers addressing the same or similar topic in Latin-america. Systematic reviews and meta-analysis have been published on the topic

**Thank you for this useful suggestion. We amended the second paragraph of the introduction to include results from systematic reviews and more information about the Latin-American literature as follows:**

**“Several recent systematic reviews have summarised the literature regarding risk factors for COVID-19 complications.[8–12] Results from these systematic reviews show that factors consistently associated with a higher risk of COVID-19 complications are pre-existing comorbidities, such as hypertension, heart failure, diabetes mellitus, coronary heart disease, cancer, chronic obstructive pulmonary disease, smoking, age and male gender.[8–10] Obesity, psychiatric conditions and pregnancy have also been associated to COVID-19 negative outcomes and severe symptoms.[5,13–18] Nevertheless, most of the evidence in these systematic reviews comes from Europe, USA and Asia, with very little research on Latin-American populations.[8–10] More recently, a large observational multinational study explored risk factors for COVID-19 complications among hospitalized patients in Latin America.[19] Risk factors independently associated with progression to ICU admission were age, shortness of breath, and obesity.[19] Another large population-based study among confirmed-COVID-19 cases in Mexico, age was the most predictive factor for mortality. Renal disease, hypertension, diabetes and obesity were as well associated with a higher risk of dead due to a COVID-19 infection.[20] Nevertheless, studies exploring risk factors for COVID-19 complications at population level are scarce.” (Page 4, lines 69-82)**

**We also added the following new references, five systematic reviews and two recent Latin-American studies:**

- **Makovski TT, Ghattas J, Monnier-Besnard S, Cavillot L, Ambrožová M, Vašinová B, et al. Multimorbidity and frailty are associated with poorer SARS-CoV-2-related outcomes: systematic review of population-based studies. Aging Clin Exp Res. 2024;36: 40. doi:10.1007/s40520-023-02685-4**
- **Li C, Islam N, Gutierrez JP, Gutiérrez-Barreto SE, Castañeda Prado A, Moolenaar RL, et al. Associations of diabetes, hypertension and obesity with COVID-19 mortality: a systematic review and meta-analysis. BMJ Glob Health. 2023;8: e012581. doi:10.1136/BMJGH-2023-012581**
- **Haber R, Ghezzawi M, Puzantian H, Haber M, Saad S, Ghandour Y, et al. Mortality risk in patients with obesity and COVID-19 infection: a systematic review and meta-analysis. Metabolism. 2024;155. doi:10.1016/j.metabol.2024.155812**
- **van Baar JAC, Kostova EB, Allotey J, Thangaratinam S, Zamora JR, Bonet M, et al. COVID-19 in pregnant women: a systematic review and meta-analysis on the risk and prevalence of pregnancy loss. Hum Reprod Update. 2024;30: 133–152. doi:10.1093/humupd/dmad030**
- **de Paula Silva-Lalucci MP, Marques DC de S, Valdés-Badilla P, Andreato LV, Magnani Branco BH. Obesity as a Risk Factor for Complications and Mortality in Individuals with SARS-CoV-2: A Systematic Review. Nutrients. 2024;16. doi:10.3390/nu16040543**
- **Reyes LF, Bastidas A, Narváez PO, Parra-Tanoux D, Fuentes Y V., Serrano-Mayorga CC, et al. Clinical characteristics, systemic complications, and in-hospital outcomes for patients with COVID-19 in Latin America. LIVEN-Covid-19 study: A prospective, multicenter, multinational, cohort study. PLoS One. 2022;17. doi:10.1371/journal.pone.0265529**
- **Liao LD, Hubbard AE, Gutierrez JP, Juárez-Flores A, Kikkawa K, Gupta R, et al. Who is most at risk of dying if infected with SARS-CoV-2? A mortality risk factor analysis using machine learning of patients with COVID-19 over time: a large population-based cohort study in Mexico. BMJ Open. 2023;13: e072436. doi:10.1136/BMJOPEN-2023-072436**

6. Explain the reasons for an invalid RUN o lack of data of enrolment

**Thank you for the opportunity to explain this. The dataset contained some RUN numbers that were not valid (n=8). It is likely that these invalid RUN numbers represent typing errors or transient enrolments. We had to exclude these cases as it was not possible to link them with hospital and national records. The Ancora Network dataset contained some records with a missing value for the date of enrolment (n=301). It is likely those records represent transient enrolments or old enrolments that were manually included in the records. We could not include those records as we used the date of enrolment to identify people in the sample that was enrolled in one of the three primary healthcare centres during the study period. We added the following sentence in the method section:**

**“People were excluded if they had an invalid National Identification Number (8 people with invalid RUN), or when no date for enrolment was available (n=301) due to errors in the dataset.” (Page 6, lines 126-128)**

7. Provide further details on the data source; why you coded the conditions? Those are not register directly there?

**Thank you for raising this issue. The Ancora Network use a clinical record software that is organised based on clinical contacts or appointments. The reason (or diagnoses) related to that clinical contact is recorded by the healthcare professional but is not systematically organised and coded. Furthermore, one clinical contact could be related to several different diagnoses. We identified chronic conditions by extracting the information on diagnoses available at every clinical contact the person had, and the information on every medication people were prescribed. We added the following explanation in the method section:**

**“As diagnoses and chronic conditions are not systematically coded in the Ancora Network clinical records, we designed a code to identify these 14 conditions in non-structured records of diagnosis available from primary care clinical records data”. (Page 7, lines 156-157)**

8. It is not clear the procedure for comorbidities, in particular what this means: Therefore, we included in the model those comorbidities with a good level of identification based on their prevalence in the sample

**Thank you for this comment. We appreciate the sentence explaining this procedure was not clear enough. In addition to the counting of comorbidities or the count of dispatched drugs, we wanted to explore the independent effect of cardiovascular and other chronic pre-existing conditions identified in the literature as risk factors for COVID-19 complications. Nevertheless, for some chronic conditions such as cerebrovascular disease or ischaemic heart disease, the number of people identified from primary care records was significantly lower than expected based on estimations from the National Health Survey. These results lead us to believe these conditions were likely to be underreported in primary care records and decided to include pre-existing conditions such as hypertension, diabetes and depression that are more likely to be well reported in primary care records. We changed that sentence and added the following explanation:**

**“We also included in the models the conditions hypertension, diabetes and depression to explore their independent effect in the outcomes. We did not include other chronic conditions as their proportion in the cohort was significantly lower than expected based on estimations from the National Health Survey and we hypothesise they were likely being underreported in the data.” (Page 10, lines 210-213)**

9. Please clarify in lines 202 & 203: those individuals were newly enrolled on that period? Are the total number of individuals enrolled? How this is a sample if you are including all?

**Thank you for highlighting this issue. We included all individuals enrolled in one of the three Ancora Network Primary Healthcare centres. We appreciate the word ˈsampleˈ could be misinterpreted in this context. Therefore, we changed the word to ˈcohortˈ in both lines and throughout the text to be consistent. (Page 11, lines 238 and 246; Page 12 line 260; Page 15, line 332, Page 19, lines 386, 394 and 398)**

10. In table 1 will be useful to add a column for those that tested positive

**Thank you for this suggestion. We added in table 1 a column with participants´ characteristics for those with a confirmed COVID diagnosis in the cohort. (Page 13).**

Reviewer #1: The authors present a study aimed to evaluate the association between demographic and clinical characteristics with severe complications by SARS-CoV-2 infection, using a retrospective population-based cohort from Chile (n=44,674).
The article is well written but, in my opinion, it requires significant changes. My specifics comments are below.

11. Major Comments
    I consider that the document lack of a clearly defined study population and study outcomes. Given that authors aimed to evaluate complications by SARS-CoV-2 infection, it is not entirely clear why no-COVID-19 subjects were also analyzed.

**Thank you for this important comment. The aim of this study was to examine risk in the general population. However, identifying people with a confirmed COVID-19 infection in the community is not possible without introducing bias in the sample. This is mainly because COVID-19 testing was not always carried out at community level, especially at the beginning of the COVID-19 Pandemic and among asymptomatic patients or patients with mild symptoms. Furthermore, there were important differences in the type of COVID-19 test used and test results recorded across healthcare settings. We had access to PCR test results at the National level linked to the Ancora Network dataset. However, we did not have access to information on Antigen Rapid test results, which were more likely to be used in the community. Therefore, we decided to include all adults enrolled in the Ancora Network Healthcare centres to avoid misclassification bias. This approach has been taken by Williamson et al in the UK. We included a paragraph explaining this decision in the “design and data sources” section as follows:**

**“The aim of this study was to examine risk in the general population rather than in a population infected with COVID-19. This approach has been taken by other researchers to avoid misclassification bias,[18] as testing for COVID-19 cases has not always been carried out at community level. Therefore, all patients were included irrespective of any COVID-19 test results.” (Page 6, lines 120-123).**

**We also added Williamson et al (2020) reference.**

12. Additionally, considering that Ancora Network population is not representative of Chilean population, it is not evident why did the authors decided to analyze these specific inhabitants.

**Thank you for this comment. While the Ancora Network population is not necessarily representative of the whole Chilean population, it provides a unique opportunity to explore the risk of COVID-19 complications at a population level. To our knowledge, this is the only healthcare service in Chile that has linked all their primary care healthcare records with hospital records and National registries such as death mortality records and COVID-19 vaccine records. We added a paragraph in the “Design and data sources” section as follows:**

**“We used this dataset as it is the only dataset in Chile that currently links primary healthcare records with hospital records and national registries such as the national immunization registry and mortality records.” (Page 5, lines 105-107)**

13. Moreover, the terms "any hospital admission", "hospital admissions due to a COVID-19", "ICU admission" and "ICU admission due to COVID-19" are mentioned along the manuscript, making it hard to follow.

**We thank the reviewer for this important comment. We agree the terms we used to refer to the outcomes can be confusing and are not consistent across the manuscript. The three main outcomes of the study (Hospital admissions, ICU admissions and death) are all specific to COVID-19 infections. We did not include in the outcomes a hospital admission or death due to other conditions. We changed the terms for the outcomes as follows:**

**- a hospital admission due to COVID-19 infection**

**- an ICU admission due to COVID-19 infection**

**- a death due to COVID-19 infection**

**We also made sure these terms were changed across the manuscript consistently.**

14. In this sense, it is important that authors bring context regarding the selection of the study population and decide to select or not only COVID-19 subjects. In the case that they decided to analyze no-COVID-19 persons, it is necessary to include an outcome regarding SARS-CoV-2 infection and develop a subgroup analysis considering this outcome.

**Thank you for this suggestion. As we explained in a previous comment, our aim was to explore the risk of COVID-19 complication at the population level. Including only people with a COVID-19 confirmed infection will introduce bias toward an overestimation of the risk at the population level. Therefore, we decided to analyse the entire cohort. However, we included as suggested in other comments, a column with COVID-19 confirmed infections in Table 1, and a subgroup analysis only for people with a COVID-19 confirmed infection (Table S8). Results from this subgroup analysis are generally consistent with the analysis for the entire cohort. Nevertheless, the subgroup analysis among people with COVID-19 confirmed infections shows higher Hazard Ratios for age and lower Hazard Ratios for COVID vaccine as we expected due to the different population risk profiles. We added the following sentence in the method:**

**“We used two subgroup analyses. The first subgroup analysis was used to explore the effect of sex and pregnancy on the outcomes of this study. As no pregnant or puerperal women died of COVID-19 in this cohort, pregnancy was excluded from the models for COVID-19 death. The second subgroup analysis was used to explore the risk of having a COVID-19 complication only among people who had a confirmed COVID-19 infection during the study period.” (Page 10, lines 222-226)**

**And a two sentences in the results section as follows:**

**“The second subgroup analysis among people with a confirmed COVID-19 infection shows similar results, with Hazard Ratio for age and COVID-19 vaccine doses higher than the Hazard Ratio in the models for the entire cohort. Sex is no longer statistically significantly associated with any of the three outcomes when including only people with a confirmed COVID-19 infection (S8 Table).” (Page 16, lines 334-337)**

15. It is also needed that authors define and homologate the study outcomes along the manuscript.

**Thank you for this useful suggestion.** **We changed the terms for the outcomes across the manuscript as follows:**

**- a hospital admission due to COVID-19 infection**

**- an ICU admission due to COVID-19 infection**

**- a death due to COVID-19 infection**

16. Given that the findings of this study are previously reported, in my opinion the novelty of the research objective was not entirely clear.

**We appreciate this comment. We agree with the reviewer the findings of our study are similar to what has been found in other studies. However, the vast majority of the evidence on risk factors for COVID-19 complications reported in the literature comes from Europe, USA and Asia, with an important dearth of articles coming from Latin-American populations. Evidence on risk factors for COVID-19 complications from Europe and USA does not necessarily apply in Latin-America. Factors such as ethnicity, epidemiological context and healthcare system differences across regions might influence the effect of sociodemographic and clinical factors on the outcomes we are analysing. Understanding to what extent findings from previous studies performed in other contexts are applicable to our populations is key for policy and healthcare provision.**

**While there are some studies exploring risk factors for COVID-19 complications in Latin American countries, most of the evidence comes from studies using small samples, in specific populations, and do not consider the effect of time in the analysis, which might overestimate the risk at a population level. To our knowledge, this is the first population-based study exploring factors associated with COVID-19 complications in Chile. Using a large sample size, a follow-up period of two years and accounting for differences in the pandemic period, we evaluate the effect of several sociodemographic and clinical factors on the risk of hospital admissions, ICU admissions and COVID-19 deaths. In this context, we believe our findings could be an important contribution to the field.**

**We appreciate this might not have been completely explained in the manuscript. We added the following sentences in the discussion section:**

**“To our knowledge, this is the first population-based study exploring factors associated with COVID-19 complications in Chile. Most of the evidence in risk factors for COVID-19 complications comes from Europe, USA and Asia, which might not necessarily be useful for the Latin American context.” (Page 18, lines 358-360)**

**“This design allowed us to access a large cohort of exposed people during the period of study, being one of the largest cohort studies exploring risk factors for COVID-19 complications, contributing the knowledge in this field for the Latin American population.” (Page 20, 419-422)**

Minor Comments

-Introduction:

17. As mentioned before please include a brief explanation of the population origin. A map with location of the three primary healthcare centers and the districts may be helpful. To bring context to readers, the introduction may also include information regarding healthcare insurance, FONASA, Ancora Network and COVID-19 epidemiological surveillance in Chile.

**Thank you for this useful suggestion. We added the following paragraph to the introduction:**

**Chile has a well-established social security system, with 98% of the population having health insurance, 77% of them affiliated to a public insurance system (FONASA).[21] Despite being ranked as the 28th country with the highest Global Health Security (GHS) Index,[22] and developed an integrated reporting system for COVID-19 surveillance,[23] Chile was the fifth Latin-American country with more COVID-19 reported cases since the beginning of the Pandemic, with and a cumulative number of 5.3 million COVID-19 infections and 61 thousand deaths by November 2023.[24] (Page 5, lines 83-88)**

**We added three new references:**

- **Aguilera X, Castillo-Laborde C, Ferrari MN De, Delgado I, Ibañez C. Monitoring and Evaluating Progress towards Universal Health Coverage in Chile. PLoS Med. 2014;11: e1001676.**
- **Bell JA, Nuzzo JB. Global Health Security Index: Advancing Collective Action and Accountability Amid Global Crisis.2021. Available: https://ghsindex.org**
- **Taramasco C, Rimassa C, Acevedo Romo J, Césped Zavando A, Fuentes Bravo R. Epidemiological surveillance in COVID- 19 pandemic: EPIVIGILA system. Medwave. 2022;22: e002560–e002560.**

**We included a map with location of the three primary healthcare centres in the supplementary material (S2_Fig)**

-Materials and Methods:

18. Please describe the calculation of follow up time and the definition of COVID-infection (it was only by PCR? Clinical definition? Antigen Rapid test was not used for epidemiological surveillance?).

**Thank you for this comment. COVID-19 cases among those who had hospital admissions were identified based on PCR test results recorded in the hospital records or from the National Covid-19 PCR surveillance data. COVID-19 deaths were identified based on ICD-10 codes in the underlying or contributing cause of death recorded in death certificates. The National Covid-19 surveillance data we accessed contained all PCR test results performed in the country during the study period and did not include Antigen Rapid Test results. To explain this, we added the following sentences to the method section:**

**“The National COVID-19 PCR surveillance data contained the results of all PCR tests performed in Chile during the study period.” (Page 5, lines 102-103)**

**“COVID-19 cases were identified among those admissions based on PCR test results recorded in the hospital records or the National Covid-19 PCR surveillance data.” (Page 7, lines 137-139)**

19. As above-mentioned and similar whti COVID-19 death, definitions of main outcomes including hospitalization and UTI may be only restricted to COVID-19 related. A combination of the three outcomes may be also considered.

**Thank you for this comment. As mentioned in response to another comment, the three main outcomes of the study (Hospital admissions, ICU admissions and death) are all COVID-19 infections related. We did not include in the outcomes a hospital admission or death due to other conditions. We changed the terms for the outcomes to avoid that confusion and added the following sentences in the method section:**

**“We only considered admissions due to a COVID-19 infection” (Page p 7, line 139)**

20. Other variables that may be helpful may be obesity, chronic kidney disease, wave of infection, mechanical ventilation, and cause of encounters with a physician or a nurse.

**We appreciate this suggestion. We agree with the reviewer those variables might be helpful to include in the analysis. Unfortunately, information regarding BMI, Chronic kidney disease and whether patients required mechanical ventilation is not available in the data and therefore could not be included. The cause of encounters with physicians is not systematically extracted from clinical records, as it is recorded as free text, and therefore it is also not possible to be included.**

**We agree including information about waves of Covid-19 infection in the Chilean Metropolitan Region might be helpful for context. We used wave period definitions from Ayala et al. (2023). We added the definition of the wave periods in the method section and an indication of the waves in figure 2 (Former Figure 1). We included a sentence in the results section summarizing the wave period where hospital admissions and deaths occurred. Nevertheless, the wave period is highly correlated with the number of Covid-19 vaccine doses. Therefore, this variable was not included in the models.**

**In summary, the following sentences were added to the main text:**

**“Based on Ayala et al,[19] we identified the Pandemic waves were hospital admissions and deaths due to COVID-19 occurred.” (Page 7, lines 144-145)**

**“Of the 455 hospital admissions identified in the cohort, 161(35.38%) occurred during the first Pandemic wave, 221(48.57%) during the second Pandemic wave, 5(1.1%) during the third Pandemic wave, and 68(14.95%) occurred between wave periods.” (Page 12, lines 262-264)**

**“Of the 148 COVID-19 deaths identified in the cohort, 68(45.95%) occurred during the first Pandemic wave, 38(25.68%) during the second Pandemic, and 42(28.38%) occurred between wave periods.” (Page 12, lines 267-269)**

21. The subgroup analysis may be sustained in background and objectives.

**Thank you for this comment. We added the following to the aim:**

**“We aimed to evaluate the association between sociodemographic, clinical factors and the risk of hospital admissions, admissions to ICU and death due to a COVID-19 infection among adults in a community-based healthcare network in Chile, and its effect by sex and COVID-19 infection.” (Page 5, lines 91-93)**

-Results and Discussion:

22. A flowchart showing the selection steps may be included as principal figure.

**Thank you for this suggestion. We included a flow diagram with the number of people included and excluded in each stage and the final number of people included and with an identification of cases for the outcomes by centre (Figure 1).**

23. Please present results regarding follow-up time.

**We appreciate this comment and apologies for missing that information. We added the mean number of days of follow-up time (and standard deviation) in table 1 for each column and added the following sentence in the text:**

**“The mean follow-up period for the entire cohort was 712.9 (SD 84.27) days.” (Page 11 lines 255-256).**

24. Figure 1: please also add a curve showing the COVID-19 cases.

**Thank you for this suggestion. We included in that figure (Figure 2 now) the number of confirmed COVID-19 cases among participants in the cohort by epidemiological week. As the number of COVID-19 cases by week is much higher than the number of hospital admissions or deaths, we added a second y-axis to make the figure easier to read.**

25. Table 1 and S5 table: please include totals in the first row and present statistical test comparing groups.

**We appreciate this suggestion. We added totals in the first row and the p-value of a chi2 test or t-test depending on the type of variable. We also added the following sentence in the analysis section:**

**“Chi2 and t-test were used to analyse associations between the outcome variables and categorical or continuous variables respectively.” (Page 9, lines 186-187)**

26. Table 2: add the variable "number of COVID-19 infections ".

**Thank you for this suggestion. As all people with the outcome had a COVID-19 infection, it is not possible to include that variable in the analysis for the entire cohort. Nevertheless, we included in table S8 the subgroup analysis only for people with a COVID-19 confirmed infection.**

27. Please correct the term "prevalence" in Line 176.

**Thank you for highlighting this error. We changed the word “prevalence” for “proportion” (page 9, line 203).**

28. Include as limitation of the study the lack of information regarding clinical conditions control such as of diabetes and hypertension.

**Thank you for this suggestion. We included the following sentence in the “strengths and limitations” section:**

**“We were not able to include all pre-existing chronic conditions, or symptoms, or diabetes and hypertension level of control due to the lack of structured and systematic recording in primary healthcare records.” (page 21, lines 426-428)**

29. -Subsidiary aspects
    Specify the A (lowest income), B ,C , and D (highest income) categories for FONASA variable in all tables

**Thank you for this useful suggestion. We added that information in both tables. (Pages 13 and 17)**

30. Reviewer #2: Interesting study
    In the summary it says: "Among the 44,674 people, 460 (1.03%) had a hospital admission, 217(0.49%) had an ICU admission and 533(1.19%) died due to COVID-19," which does not apply completely with the results. I suggest writing it as it is in the results... 217 of them were also admitted to the ICU (xxx%).

**Thank you for this suggestion. We changed that sentences to include the suggestion as follows:**

**“Among the 44,674 people, 455 (1.03%) had a hospital admission due to a COVID-19 infection and 2167(0.49%) of them also had an ICU admission.” (Page 2, lines 39-40)**

31. "In addition, the death toll must be changed from 533, which corresponds to the total cohort, to 89, which are those who died due to COVID-19."

**Thank you for highlighting this issue. The 533 include all-cause deaths for the entire cohort. The 89 correspond to COVID-19 deaths among those who were admitted to hospital. The number of people who died due to a COVID-19 infection in the entire cohort was 148. Apologies if this was not clear. We changed that sentence accordingly as follows:**

**“Among the 44,674 people, 148(0.33%) died due to COVID-19 infection.” (Page 2, line 41)**

32. In the summary it says: "Hypertension and diabetes pre-existing conditions … " I would eliminate "pre-existing conditions" because it is obvious that they are pre-existing.

**Thank you for this suggestion. We removed the term “pre-existing conditions” and the sentence reads as follows:**

**“Hypertension and diabetes were associated with a higher risk of a hospital admission and death, but not with an ICU admission due to COVID-19 infection.” (Page 2, lines 42-43)**

33. The expression "any hospital admission" is also not clear; "hospital admission" would be better.

**Thank you for highlighting this issue. We agree with both reviewers the terms used to describe the outcomes were not clear and consistent across the manuscript. We changed the terms for the outcomes as follows:**

**- a hospital admission due to COVID-19 infection**

**- an ICU admission due to COVID-19 infection**

**- a death due to COVID-19 infection**

**We also made sure these terms were changed across the manuscript consistently.**

34. Also, in the summary it says: "from higher-income", it should say "from higher-income countries".

**Thank you for finding this error. We added the word “countries” to that sentence in the conclusions. (Page 3, line 51)**

35. Among the authors' workplaces appears: "(6) (School of Computing and Information Systems, University of Melbourne)", however none of the authors have that membership.

**We apologies for that error. We added the affiliation accordingly. (Page 1, line 6)**

36. It would be useful to have a table comparing the risk factors of all COVID patients with those who die from COVID, not just the entire cohort as shown in table 1.

**Thank you for this useful suggestion. We added a column with characteristics for people with a confirmed COVID-19 infection in table 1. (Page 13)**

37. In Sociodemographic factors it says: "People 70 years or older had 1.51 times (HR 1.51; 95% CI 1.42 to 1.62) and 38% 240 (HR 1.38; 95% CI 1.24 to 1.53) higher risk ..". I think it is better to keep the same numerical expression format, this is 1.51 times, without mixing it with percentages.

**Thank you for this suggestion. We changed it as follow:**

**“People 70 years or older had 1.53 times (HR 1.53; 95% CI 1.43 to 1.64) and 1.40 (HR 1.40; 95% CI 1.24 to 1.55) higher risk of being admitted to hospital or have an ICU admission due to a COVID-19 infection than those between 18 and 34 years old respectively during the study period.” (Page 14, lines 289-290)**

38. It says: "People 70 years or older had 1.86 times (HR 1.86; 95% CI 1.68 to 2.06) higher risk of dying due to a COVID-19 … than those aged 54 years or younger." However, the basis of comparison according to table 2 is with ages between 18 to 34.

**Thank you for highlighting this issue. For the outcome death due to COVID-19 infection, we regroup the categories 18-34 and 35-54 yo as no COVID-19 deaths were observed in the younger age category group. Therefore, the reference category for that analysis was 18-54 yo. We added a note in the table 2 to clarify. (Page 17, line 343)**

39. It says: "Compared with male sex, females were 0.96, 0.94 and 0.68 times less likely .. l", the confidence intervals are missing.

**Thank you for this suggestion. We added the confidence intervals to the estimates in that sentence as follows:**

**“Compared with male sex, females were 0.96 (95% CI 0.93 to 1.00), 0.94 (95% CI 0.89 to 0.99) and 0.64 (95% CI 0.45 to 0.91) times less likely to be admitted to hospital, admitted to ICU or die due to a COVID-19 infection (Table 2 and Figure 2).” (Page 14, lines 294-295)**

40. The title of table number 2 says: "Hazard Ratio of the risk for any hospital admission or an ICU admission due to …… ." "COVID deaths" must be included

**Thank you for finding this error. We changed the title of table 2 to address comments regarding the terms we used for the outcome variables and added the outcome death due to COVID-19 infections as follows:**

**“Table 2. Hazard Ratio of the risk for a hospital admission, an ICU admission or death due to COVID-19 infection between 2020 and 2021.” (Page 17, lines 341-342)**

41. In Clinical factors it says: "The number of frequently dispatched drugs was associated with a higher risk of hospital and ICU admissions … " Must include numbers.

**Thank you for this suggestion. We added the Hazard Ratio and 95% confident intervals´ values as follows:**

**“The number of frequently dispatched drugs was associated with a higher risk of a hospital (HR 1.10; 95% CI 1.07 to 1.14) and ICU admission (HR 1.13; 95% CI 1.08 to 1.17) due to COVID-19 infection but not with death due to COVID-19 infection (HR 1.03; 95% CI 0.98 to 1.08).” (Page 14, lines 304-306)**
