# Supplementary material for: Association between demographic, clinical characteristics and severe complications by SARS-CoV-2 infection in a community-based healthcare network in Chile
Source: PLoS One. 2024 Dec 30;19(12):e0314376. doi: 10.1371/journal.pone.0314376 (PMC11684639; doi:10.1371/journal.pone.0314376)
Supplement: Attachment — Submitted filename: Responses 2.docx [file pone.0314376.s010.docx]

**Association between demographic, clinical characteristics and severe complications by SARS-CoV-2 infection in a community-based healthcare network in Chile**

**Thank you for the opportunity to respond to the peer reviewers’ comments. We hope we have addressed all the comments. Our responses are included below.**

Journal Requirements:

Please review your reference list to ensure that it is complete and correct. If you have cited papers that have been retracted, please include the rationale for doing so in the manuscript text, or remove these references and replace them with relevant current references. Any changes to the reference list should be mentioned in the rebuttal letter that accompanies your revised manuscript. If you need to cite a retracted article, indicate the article’s retracted status in the References list and also include a citation and full reference for the retraction notice.

**Apologies for missing this error. We removed the following references that were reported as retracted:**

- **Giannouchos T V. et al. doi:10.1183/13993003.02144-2020 (Page 23, lines 536-540 tracked-changes version)**
- **Risk factors for mortality in patients with Coronavirus Disease 2019 (COVID-19) in Bolivia: An analysis of the first 107 confirmed cases (Page 24, lines 560-562 tracked-changes version)**

**And we updated the following references:**

- **Treskova-Schwarzbach M, et al. 2021. doi:10.2139/SSRN.3791433. In this case, we included the published version, as the previous one was a pre-print version. (Page 21, lines 472-473 tracked-changes version)**
- **INE. Resultados Censo 2017. 2019. In this case, we added the url and date of access. (Page 22. Lines 512-513)**

Review Comments to the Author

Reviewer #1: The authors had made changes improving considerably their manuscript.

**Thank you for this supportive comment.**

However, we still have the following suggestions:

-Please add information regarding COVID-19 epidemiological surveillance in Chile. Given that only PCR information was analyzed, it is important to clarify why Antigen Rapid Test data was not considered. COVID-19 epidemiological surveillance in Chile was only based on PCR? Was Antigen Rapid Test data not available for analysis? If the last case is true, this lack of availability may be pointed as a very important limitation of the report.

**Thank you for this comment. Antigen Rapid Test was available for surveillance in Chile but not from the beginning of the Pandemic. It was considered in the definition for a COVID-19 confirmed case only from 2021. The National surveillance data included all confirmed and reported COVID-19 cases, but case definition changed was not consistent across this period. Also, as we explain later, confirmed cases do not represent all COVID-19 cases during this period and less severe or asymptomatic cases are more likely to be missed than more severe cases. We agree this was not clearly explained. We included the following sentence in the method and limitation sections:**

***“The national COVID-19 PCR surveillance data contained information on all COVID-19 confirmed cases in Chile during the study period, with included PCR and Antigen Rapid Test confirmed cases”. (Page 5, lines 102-104 in tracked-changes version)***

***“Nevertheless, including only people with a positive COVID-19 PCR or Antigen Rapid Test would have sub estimate the number of people infected during this period” (Page 20, line 427 in tracked-changes version)***

- The last paragraph in the introduction section benefits from a general aim that encompasses all the analysis and subgroups analysis carried out. This aim may be split into specific objectives.

**Thank you for this suggestion. We made the following changes:**

***“We aimed to evaluate the association between sociodemographic, clinical factors and the risk of COVID-19 complications among adults from a community-based healthcare network in Chile. We evaluated the association between sociodemographic, clinical factors and the risk of hospital admissions, admissions to ICU and death due to a COVID-19 infection, and estimated the effect by sex and COVID-19 infection in these associations.” (Page 5, lines 89-93 in tracked-changes version)***

- The methodology section requires an explanation regarding calculation of follow up time.

**Thank you for this suggestion. We added the following sentence in the analysis section:**

***“We calculated the follow-up time based on the date the participant was registered in the healthcare centre or the study starting date (1st January 2020) whichever was later, and the date of the event (hospital admission or death) or the end of the study period (31st December 2021) whichever was first.” (Page 9, lines 192-194 in tracked-changes version)***

- Table 2: please revise the title which started as "Fig 4".

**Thank you for noticing this error. We added the space between the title of figure 4 and table 2. (page 16, line 336 in tracked-changes version*)***

Is the "n=42,588" correct? It is different from "n=44,674" on table 1.

**Thank you for raising this issue. As the variable “FONASA” has 2082 missing values, those participants the model removes those participants from the analysis. As we indicated in page 10 line 213, missing values were excluded from the model as the proportion was small (<5%).**

**Regardless, 4 participants were wrongly coded as having a cero following time and therefore removed from the Cox regression model. We rectified that error in the data and ran the models again. This did not change the results, so tables were not amended except for the n that was changed to 42,592 in table 2; and 18,496 and 24,096 for males and females respectively in table S7.**

If the model is considering COVID-19 and non-COVID-19 cases, why is not possible to calculate HR for COVID-19 infection as outcome?

**Thank you for this question. We can calculate the HR for COVID-19 infection indeed. What we meant previously is that including the variable COVID-19 infection in models for any hospital admission or death due to a COVID-19 infection was not possible as all participants with a hospital admission due to COVID-19 or COVID-19 death were COVID-19 infected patients as well. Apologies if our former explanation was not clear.**

**We explicitly decided not to include COVID-19 infection as an additional outcome in our study because it was not the aim of the study to estimate the risk of COVID-19 infection but the risk of COVID-19 complications. Also, we cannot be certain all COVID-19 cases in the community were properly identified, except in hospital or decedents. It is very unlikely that a COVID-19 case was not identified in hospital or mortality records, as patients were regularly tested in these settings. But it is very likely COVID-19 case were not identified in the community setting. Therefore, calculating the risk of a COVID-19 infection at a population level would underestimate the risk, as the COVID-19 infections identified in our data are likely to represent more severe cases.**

- The lack of availability of other variables such as obesity, chronic kidney disease, wave of infection, mechanical ventilation may be included into the study limitations.

**We appreciate this suggestion. We added the following sentence in the strengths and limitation section:**

**“*We were not able to include all pre-existing chronic conditions associated with COVID-19 infection in the literature, such as obesity, chronic kidney disease, symptoms, diabetes and hypertension level of control, or factors such as mechanical ventilation or wave of infection due to the lack of structured and systematic recording in primary and hospital healthcare records*.” (Page 19, lines 419-421)**

-Specify the A (lowest income), B, C , and D (highest income) categories for FONASA variable in all supplementary tables and figures (S1_Table.docx, S4_Fig.docx, S5_Table.docx, S7_Table.docx).

**Thank you for this comment. We amended the tables and figures as requested.**
